# Characterization of Fungal Pathogens Causing Blueberry Fruit Rot Disease in China

**DOI:** 10.3390/pathogens14020201

**Published:** 2025-02-18

**Authors:** Yueyan Zhou, Wei Zhang, Linna Wu, Pengzhao Chen, Xinghong Li, Guangqin Wen, Khanobporn Tangtrakulwanich, Kandawatte Wedaralalage Thilini Chethana, Fatimah Al-Otibi, Kevin D. Hyde, Jiye Yan

**Affiliations:** 1 Beijing Key Laboratory of Environment Friendly Management on Fruit Diseases and Pests in North China, Institute of Plant Protection, Beijing Academy of Agriculture and Forestry Sciences, Beijing 100097, China; 6471105009@lamduan.mfu.ac.th (Y.Z.); zhangwei@baafs.net.cn (W.Z.); guiwawa4567@outlook.com (L.W.); ppchen9609@outlook.com (P.C.); lixinghong@baafs.net.cn (X.L.); 2 Center of Excellence in Fungal Research, Mae Fah Luang University, Chiang Rai 57100, Thailand; kdhyde3@gmail.com; 3 School of Science, Mae Fah Luang University, Chiang Rai 57100, Thailand; khanobporn.tan@mfu.ac.th; 4 Botanical Garden of Guizhou Province, Guiyang 550025, China; lanmei8588@126.com; 5 Department of Botany and Microbiology, College of Science, King Saud University, P.O. Box 22452, Riyadh 11495, Saudi Arabia; falotibi@ksu.edu.sa

**Keywords:** *Vaccinium* spp., new host records, phylogenetic analysis, pathogenicity

## Abstract

Blueberry has been a burgeoning fruit in China in recent years, but its perishable nature places a constant strain on industrial development. To determine the pathogens infecting blueberry fruits, diseased samples were collected from Guizhou and Fujian Provinces. Isolates from the samples were identified by morphological characterization and phylogenetic analyses. Pathogenicity assays were conducted on fresh blueberry fruits using spore suspensions. Sixteen isolates were identified as seven species, namely, *Botryosphaeria dothidea*, *Botrytis cinerea*, *Cladosporium guizhouense*, *Colletotrichum fioriniae*, *Diaporthe anacardii*, *Fusarium annulatum*, and *Neopestalotiopsis surinamensis*, and their pathogenicity on blueberry fruits were confirmed following Koch’s postulates. The current study reported *Cladosporium guizhouense*, *Fusarium annulatum*, and *Neopestalotiopsis surinamensis* for the first time on blueberry. The study (1) demonstrated that fruit rot disease results from a mixed infection of multiple pathogens; and (2) expanded the understanding of causal agents of blueberry fruit rot during the growth stage, highlighting their potential as latent pathogens that contribute to post-harvest losses. Relevant results provide a reference for the etiological research and disease management in blueberry fruit diseases.

## 1. Introduction

Blueberry (*Vaccinium* spp.) has been cultivated for more than 100 years, and the industry has developed rapidly in the recent years, with the global production more than doubling in the last decade (U.S. Department of Agriculture 2021). As of 2023, the global cultivation area of blueberry comes up to 262,417 hectares, with a global production of 1.78 million tons, and China is ranked first, producing 0.56 million tons (International Blueberry Organization 2024). Blueberries are recognized as a “superfruit” for their rich nutrients, including vitamins (C and K), minerals (calcium, iron, magnesium, manganese, and zinc), dietary fiber (2.4–3.5% of fruit weight), and polyphenols (anthocyanins and flavonols) [1]. Specifically, anthocyanin flavonoids, which account for up to 60% of the total polyphenolics in ripe blueberries, may make the greatest contribution to the health benefits of blueberry in reducing the risk of cardiovascular disease, death, and type 2 diabetes, as well as improving weight maintenance and neuroprotection [2].

However, blueberry fruits are perishable as they are susceptible to mechanical damage and infectious diseases [3]. Fruit rot diseases caused by fungi, especially Botrytis fruit rot and anthracnose fruit rot, significantly influence the yield and fruit quality pre- and post-harvest [4]. *Botrytis cinerea* is the predominant causal agent of Botrytis fruit rot, causing blights of flowers, leaves, twigs and decay of fruits, also known as grey mould. Diseased fruit soft rot and develop grey mycelium [5,6]. Alongside *B. cinerea*, *B. pseudocinerea* and *B. californica* have also been reported to cause grey mould on blueberries [7,8]. Anthracnose fruit rot is caused by *Colletotrichum* spp., mainly *C. acutatum*, *C. gloeosporioides*, and *C. fioriniae* [9,10]. These pathogens can infect blueberries from flowering to harvest and can also be present latent infection and show symptoms in post-harvest period. Diseased fruits show sunken lesions with pink or salmon conidial masses [6]. Anthracnose fruit rot has been reported to cause 10–20% yield loss, and the loss caused by Botrytis fruit rot can reach 30–40% [11]. Another fruit rot, caused by *Alternaria* spp., also named Alternaria fruit rot, commonly occurs as an important post-harvest disease during transport and storage, characterized by sunkenness with white-to-olivaceous mycelium [4,6]. In addition, *Monilinia vaccinii-corymbosi* and *Exobasidium maculosum* infect young fruits in the early season, causing mummy berry and Exobasidium fruit spots, respectively [12,13]. And there are also minor blueberry fruit diseases without specific names, such as the fruit rots caused by *Diaporthe vaccinii*, *Pestalotia vaccinii*, *Phyllosticta vaccinii*, and some yeast species [4]. Although diseases caused by specific pathogens have different names, a single plant can be infected by multiple pathogens. Since most fruit diseases lead to rotting fruit, they are collectively referred to as fruit rot disease [4,11]. However, the use of specific names, like Botrytis fruit rot, Alternaria fruit rot, or anthracnose rot, helps in identifying the most effective management strategies against each particular fungal pathogen. The objectives of the present study are to identify and characterize the fungal species associated with fruit rot disease in China through morphology and phylogeny and to evaluate their pathogenicity on blueberry through pathogenicity assays, which provide a theoretical basis for future prevention and control.

## 2. Materials and Methods

### 2.1. Sampling and Isolation

Fruit rot samples were collected from blueberry orchards in Fujian and Guizhou Provinces, China, and placed in zip lock bags with tissue papers to keep the moisture. Diseased fruits were cut into 5 mm × 5 mm pieces using a sterilized blade, disinfected with 75% ethyl alcohol for 30 s, 2% sodium hypochlorite for 2 min, and rinsed in sterile water three times then dried on sterilized filter paper. Tissues were transferred onto PDA plates, cultured in a 25 °C incubator for 5 days, and mycelia grown from the tissues were sub-cultured onto fresh PDA plates. To obtain the pure culture, single spore isolation was conducted after the sporulation of the colonies. Pure cultures were saved in tubes with a PDA slant after five days.

### 2.2. Morphology Characterization

To observe the morphology of the colony, 4 mm mycelium plugs from the edge of 5-day-old cultures were inoculated on the center of PDA plates, incubated at 25 °C with 12 h light/dark cycle until they reached the margin or more than 2/3 of the plate diameter. Colonies were photographed and diameters were measured. To induce sporulation, mycelia were inoculated on CLA (carnation leaf agar) medium and PNA (SNA with sterilized pine needle) medium. Micro morphology was observed using an Axio Imager Z2 photographic microscope (Carl Zeiss Microscopy, Oberkochen, Germany) and measured with ZEN Pro 2012.

### 2.3. DNA Extraction and PCR Amplification

Mycelia of the pure culture were collected and transferred into 1.5 mL centrifuge tubes, and genomic DNA was extracted using TIANcombi DNA Lyse and Det PCR Kit (Tiangen Biotech Co., Ltd., Beijing, China). Gene regions corresponding to different genera were amplified in a C1000 TouchTM thermal cycler (Bio-Rad Laboratories Inc., Hercules, CA, USA) with a 50 μL reaction system, comprising 45 μL of Golden Star T6 Super PCR mix (1.1×) (Tsingke Biotechnology Co., Ltd., Beijing, China), 2 μL of forward and reverse primers, and 1 μL of genomic DNA. The PCR conditions were as follows: initial denaturation for 2 min at 98 °C, followed by 34 cycles of denaturation for 10 s at 98 °C, 15 s of annealing and 1 min elongation at 72 °C, and a final extension for 5 min at 72 °C. Primer sequences and annealing temperatures are listed in Table 1. PCR products were checked using 1.2% agarose gel electrophoresis under ultraviolet light of GelDocXR+ (Bio-Rad Laboratories Inc., Hercules, CA, USA) and sequenced by Sino Geno Max Co., Ltd. (Beijing, China). At first, resulting sequences of the internal transcribed spacer (ITS) region were compared with sequences in GenBank of the National Center for Biotechnology Information (NCBI) using the BLAST tool (https://blast.ncbi.nlm.nih.gov/Blast.cgi). Other gene loci were then amplified according to the resulting genus. Primers used for the PCR reactions of each fungal genus are showed in Table 2.

### 2.4. Phylogenetic Analysis

To confirm sequence quality, the chromatograms of the obtained sequences were checked using BioEdit v7.0.9.0. [32]. According to the BLAST result, the sequences of the species within the genus were downloaded from the National Center for Biotechnology Information (NCBI) with GenBank numbers. Reference sequences and sequences generated in this study were aligned using MAFFT version 7 (http://mafft.cbrc.jp/alignment/server/) [33] and adjusted manually using BioEdit v7.0.9.0. where necessary, and sequences of different gene regions were trimmed by trimAl [34] and combined using BioEdit v7.0.9.0. Concatenated gene sequences of each genus were analyzed using maximum likelihood (ML) and Bayesian inference (BI) analysis. The evolutionary models for ML and Bayesian analyses were selected using MrModeltest v. 3.7. ML analysis was conducted using the CIPRES Science Gateway (https://www.phylo.org/portal2) [35] with the RAxML-HPC2 tool on ACCESS (8.2.12) [36,37]. The GTR + I + G model for nucleotide substitutions was selected, and 1000 replicates were run to obtain bootstrap values. Bayesian posterior probabilities (BYPP) were evaluated using MrBayes v3.2.7 based on the Markov Chain Monte Carlo sampling (BMCMC) method [38]. Six simultaneous Markov chains were run for 2,000,000 generations, and trees were sampled at every 1000th generation. Phylograms were visualized with FigTree v1.4.0 [39] and edited with Microsoft Office PowerPoint 2021 and Adobe Illustrator 2020. The accession numbers of the sequences used for phylogenetic analysis and sequences generated in this study are shown in Table S1.

### 2.5. Pathogenicity Assay

Pathogenicity tests were conducted on detached fruits using spore suspension of representative isolates. Conidia were collected from fruiting bodies on the cultures, dispersed in sterile water, and adjusted to 1 × 10^6^ conidia per mL using a hemocytometer, and 0.02% tween-20 was added to increase surface activity. Fresh, healthy, and unwounded blueberry fruits were surface-disinfested in 75% ethyl alcohol for 2 min, rinsed three times with sterile water, and then air-dried on filter paper. Fruits were placed in the bottom of 9 mm petri dishes with moist filter paper and inoculated with 20 μL of conidial suspension on the fruit. The experiment was conducted on eight fruits for each isolate and repeated three times. The same amount of sterile water was dropped on the fruits as the control. Plates were placed in the plastic box covered with plastic wrap too maintain the humidity and incubated at 25 °C. Inoculated fruits were observed daily until they showed symptoms and photographed by camera and stereoscope. To fulfil Koch’s postulate, fungi were re-isolated from the diseased site and identified by morphological characters.

## 3. Results

### 3.1. Disease Symptoms and Fungal Isolation

**Figure 1:**
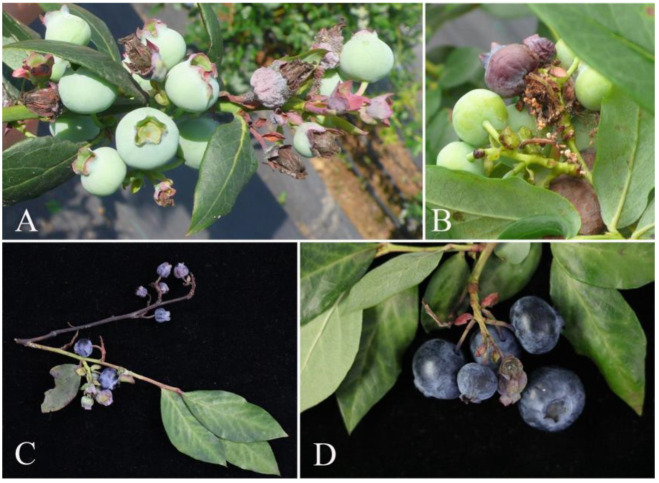
Symptoms of the blueberry fruit rot: (**A**,**B**) field symptoms of blueberry fruit rot; (**C**) died twigs with shrunken fruits; (**D**) shrivelled fruit with mould.

Symptoms appeared during the green fruit stage, showing the gradual progression of decay. Diseased fruits dried-up, shrank, rotted, and sometimes exuded fluid. Stems turned brown, dried-out, and developed mould (Figure 1). The disease incidence was 2–3%.

Sixteen isolates were generated from six samples and were identified as seven species based on morphological characters and phylogenetic analyses. They include *Botryosphaeria dothidea*, *Botrytis cinerea*, *Cladosporium guizhouense*, *Colletotrichum fioriniae*, *Diaporthe anacardii*, *Fusarium annulatum*, and *Neopestalotiopsis surinamensis*.

### 3.2. Taxonomy

***Botryosphaeria dothidea*** (Moug.: Fr.) Ces. and De Not., Comm. Soc. crittog. Ital. 1 (fasc. 4): 212 (1863).

Index Fungorum: IF 183247; Facesoffungi Number: FoF 03512.

Classification: Botryosphaeriaceae, Botryosphaeriales, Incertae sedis, Dothideomycetes, Ascomycota, Fungi [40].

*Pathogenic* on fruits of *Vaccinium* sp. **Sexual morph**: Not observed. **Asexual morph**: *Conidiomata* produced on the PNA, pycnidial, solitary, globose, dark brown. *Conidiophores*: 7–20 × 2–5 μm ($\bar{x}$ = 13.3 × 3.1 μm, n = 30), hyaline, smooth, cylindrical, or reduced to conidiogenous cells. *Conidiogenous cells*: holoblastic, hyaline, subcylindrical. *Conidia*: 19–31 × 5–9 μm ($\bar{x}\;$ = 25.2 × 6.9 μm, n = 50), hyaline, smooth with granular contents, aseptate, narrowly fusiform, with subtruncate to bluntly rounded base, apex sub-obtuse.

Culture characteristics: Colonies on PDA reaching 76 mm diam. after three days at 25 °C, initially white, aerial mycelia fluffy with an irregular margin, subsequently becoming grey, reverse black, mycelial mat moderately dense.

Material examined: CHINA, Fujian Province, isolated from diseased fruit of *Vaccinium* spp., May 2023, Y. Y. Zhou and X. H. Li (dry cultures JZBH310277, JZBH310278), living cultures JZB310277, JZB310278.

**Figure 2:**
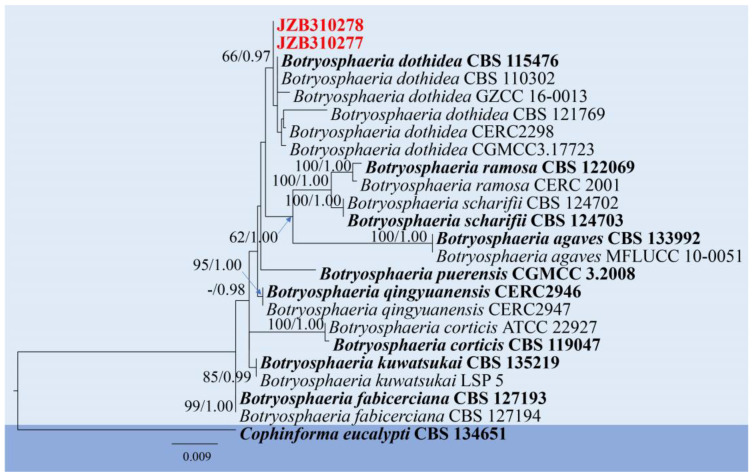
Phylogenetic tree generated by maximum likelihood (ML) analysis of combined ITS, *tef 1-α,* and *β-tub* sequence data of *Botryosphaeria* species. The tree is rooted with *Cophinforma eucalypti* (CBS 134651). The matrix has 201 distinct alignment patterns, with 15.63% being undetermined characters or gaps. Estimated base frequencies are as follows: A = 0.211405; C = 0.307730; G = 0.252687; T = 0.228178. Substitution rates: AC = 0.564762; AG = 3.067476; AT = 0.831148; CG = 0.764470; CT = 5.328705; GT = 1.000000. Gamma distribution shape parameter: α = 1.261108. ML bootstrap support values ≥ 60% and Bayesian posterior probabilities (BYPP) ≥ 0.90 are given near the nodes. The scale bar indicates 0.009 changes. The arrowhead points toward the node to which the value belongs. The ex-type strains are in bold, and newly generated sequences are in red.

**Figure 3:**
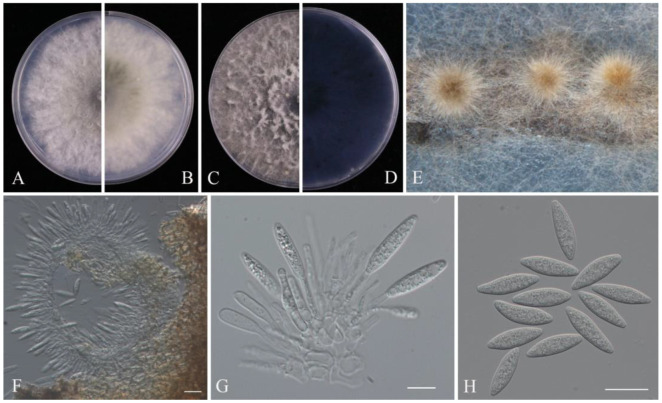
*Botryosphaeria dothidea* (JZB310278): (**A**) upper view of the colony on PDA after three days; (**B**) reverse view of the colony on PDA after three days; (**C**) upper view of the colony on PDA after seven days; (**D**) reverse view of the colony on PDA after seven days; (**E**) colony sporulating on PNA; (**F**,**G**) conidiogenous cells and developing conidia; (**H**) conidia. Scale bars: (**F**,**H**) = 20 μm; (**G**) = 10 μm.

Notes: As the type-species of *Botryosphaeria* and one of the most common species in the order Botryosphaeriales, the taxonomy of *Botryosphaeria dothidea* has undergone large changes [41]. The development of molecular technology resolved the problem of species concept among numerous morphologically similar specimens [42]. Subsequently, some taxa were separated from *B. dothidea*, and some were reduced to synonymy with *B. dothidea* [25,41]. In this study, we follow the taxonomy of Zhang et al. [25]. In the phylogenetic analyses, the two isolates (JZB310277 and JZB310278) clustered within the clade of *Botryosphaeria dothidea* with 66% ML bootstrap and 0.97 Bayesian probabilities (Figure 2), and they showed similar morphology (Figure 3) compared with the type-description of *B. dothidea* (CBS 115476) [43]. *Botryosphaeria dothidea* is globally distributed, with a wide range of woody hosts, causing canker or dieback of twigs, branches and stems, fruit rots, and even death of the plant in severe cases [41]. In China, fruit rots caused by *Botryosphaeria dothidea* have been observed on kiwifruit, plum, pomegranate, and blueberry [44,45,46,47].

***Botrytis cinerea*** Pers., Syn. meth. fung. (Göttingen) 2: 690 (1801).

Index Fungorum: IF 217312; Facesoffungi Number: FoF 03832.

Classification: Sclerotiniaceae, Helotiales, Leotiomycetidae, Leotiomycetes, Ascomycota, Fungi [40].

*Pathogenic* on fruits of *Vaccinium* sp. For morphology, see the taxonomy description by Fillinger and Elad [48] (description and illustration) and Bell et al. [6] (record on blueberry fruit).

Material examined: CHINA, Guizhou Province, isolated from diseased fruits of *Vaccinium* spp., May 2023, Y. Y. Zhou and X. H. Li (dry cultures JZBH350048–JZBH350051), living cultures JZB350048–JZB350051.

**Figure 4:**
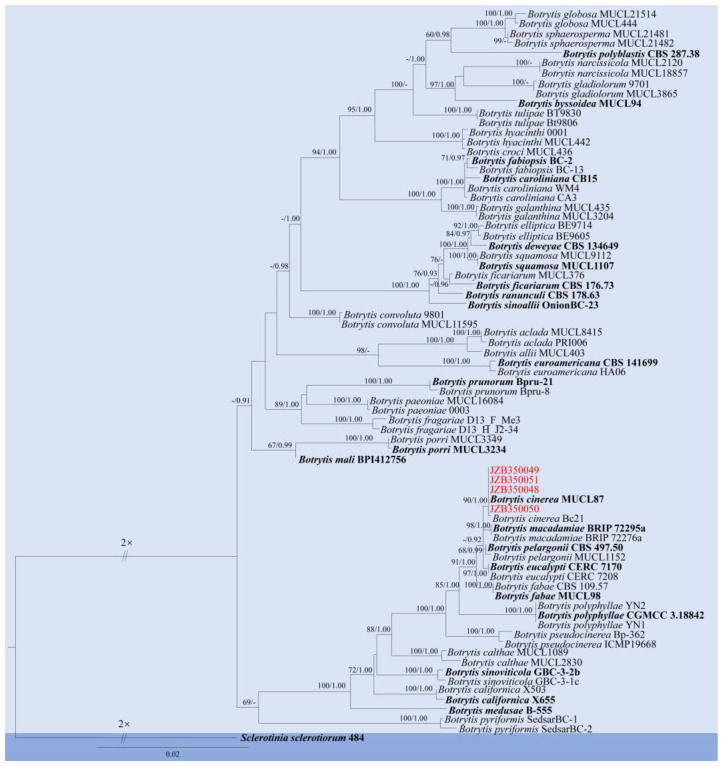
Phylogenetic tree generated by maximum likelihood (ML) analysis of combined *rpb2*, *gapdh*, and *hsp60* sequence data of *Botrytis* species. The tree is rooted with *Sclerotinia sclerotiorum* (484). The matrix has 700 distinct alignment patterns, with 2.36% of characters or gaps undetermined. Estimated base frequencies are as follows: A = 0.271469; C = 0.237560; G = 0.235321; T = 0.255650. Substitution rates: AC = 1.400180; AG = 4.179758; AT = 1.216816; CG = 0.677408; CT = 10.830994; GT = 1.000000. Gamma distribution shape parameter: α = 0.770821. ML bootstrap support values ≥ 60% and Bayesian posterior probabilities (BYPP) ≥ 0.90 are given near the nodes. The scale bar indicates 0.02 changes. The ex-type strains are in bold, and newly generated sequences are in red.

**Figure 5:**
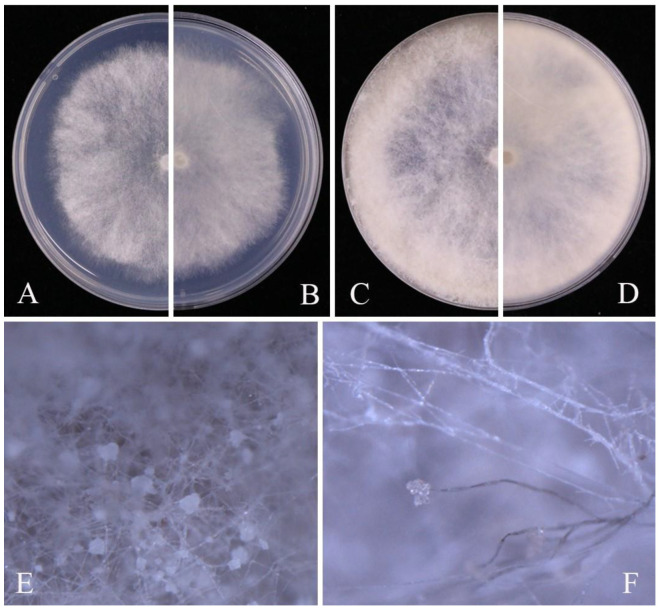
*Botrytis cinerea* (JZB350048): (**A**) upper view of the colony on PDA after three days; (**B**) reverse view of the colony on PDA after three days; (**C**) upper view of the colony on PDA after seven days; (**D**) reverse view of the colony on PDA after seven days; (**E**) mycelia; (**F**) sporulation of *B. cinerea*.

Notes: Phylogenetic analyses of the combined multi-locus dataset shows that our four isolates (JZB350048–JZB350051) clustered together with the ex-type-strain of *B. cinerea* (MUCL87), with 90% ML bootstrap values and 1.00 Bayesian probabilities (Figure 4), and the isolates are morphologically identical to *Botrytis cinerea* (Figure 5). *Botrytis cinerea* is a cosmopolitan pathogen, infecting more than 200 plant species, including fruits, vegetables, and ornamental plants, leading to significant losses [49]. The species has been reported as a major pathogen causing pre- and post-harvest fruit rot on blueberry [4].

***Cladosporium guizhouense*** S.Y. Wang, Yong Wang bis and Yan Li, MycoKeys 91: 160 (2021).

Index Fungorum: IF 842407; Facesoffungi Number: FoF 15881.

Classification: Cladosporiaceae, Cladosporiales, Dothideomycetidae, Dothideomycetes, Ascomycota, Fungi [40].

*Pathogenic* on fruits of *Vaccinium* sp. **Sexual morph**: Not observed. **Asexual morph**: Hyphomycetous. *Mycelium*: 1.7–4.2 μm wide, abundant, immersed, composed of septate, branched, pale olivaceous brown hyphae, smooth to slightly verruculose, with thin walls. *Conidiophores*: 48–112 × 3–4 μm ($\bar{x}\;$= 88.2 × 3.2 μm; n = 10), micro- to macronematous, solitary, formed laterally or terminally from hyphae, erect, branched, pale to medium olivaceous brown. *Conidia*: 3–8 × 2–4 μm ($\bar{x}\;$= 5.4 × 2.8 μm; n = 30), forming unbranched or branched acropetal chains, aseptate, pale olivaceous, smooth, thin-walled, ovoid, ellipsoid or subcylindrical. *Secondary ramoconidia* 6–20 × 3–5 μm ($\bar{x}\;$= 12 × 3.5 μm; n = 30), aseptate, pale olivaceous, smooth, thin-walled, ellipsoid to subcylindrical.

Culture characteristics: Colonies on PDA reaching 39 mm diam. after seven days at 25 °C, flat, aerial mycelium velvety to floccose, olivaceous, reverse dark grey with white margin.

Material examined: CHINA, Guizhou Province, isolated from diseased fruits of *Vaccinium* spp., May 2023, Y. Y. Zhou and X. H. Li (dry cultures JZBH390091, JZBH390092), living cultures JZB390091, JZB390092.

**Figure 6:**
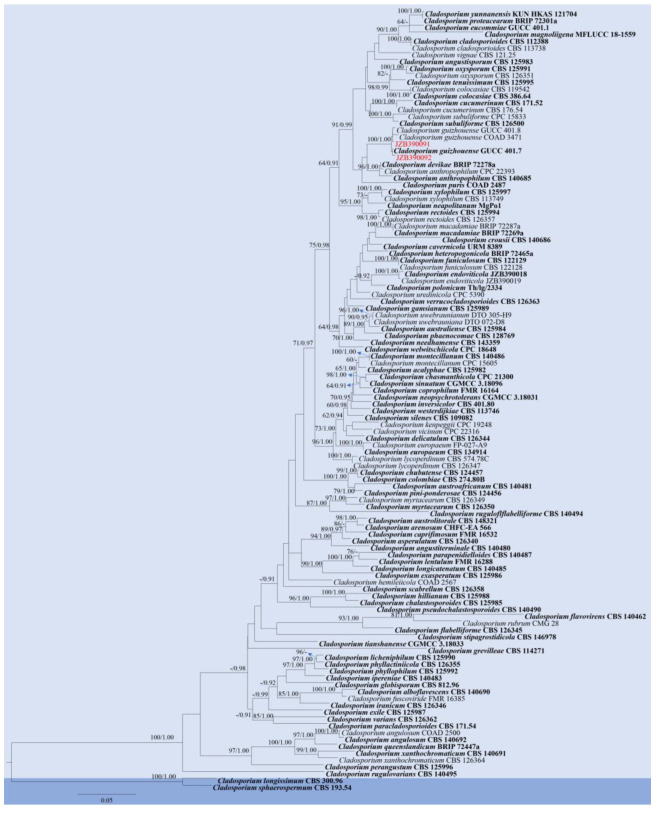
Phylogenetic tree generated by maximum likelihood (ML) analysis of combined ITS, *act*, and *tef 1-α* sequence data of *Cladosporium cladosporioides* species complex. The tree is rooted with *Cladosporium longissimum* (CBS 300.96) and *Cladosporium sphaerospermum* (CBS 193.54). The matrix has 480 distinct alignment patterns, with 6.11% of characters or gaps undetermined. Estimated base frequencies are as follows: A = 0.228323; C = 0.293145; G = 0.245258; T = 0.233274. Substitution rates: AC = 2.384560; AG = 5.066431; AT = 2.461368; CG = 1.411300; CT = 8.523585; GT = 1.000000. Gamma distribution shape parameter: α = 0.714986. ML bootstrap support values ≥ 60%, and Bayesian posterior probabilities (BYPP) ≥ 0.90 are given near the nodes. The scale bar indicates 0.05 changes. The arrowhead points toward the node to which the value belongs. The ex-type strains are in bold, and newly generated sequences are in red.

**Figure 7:**
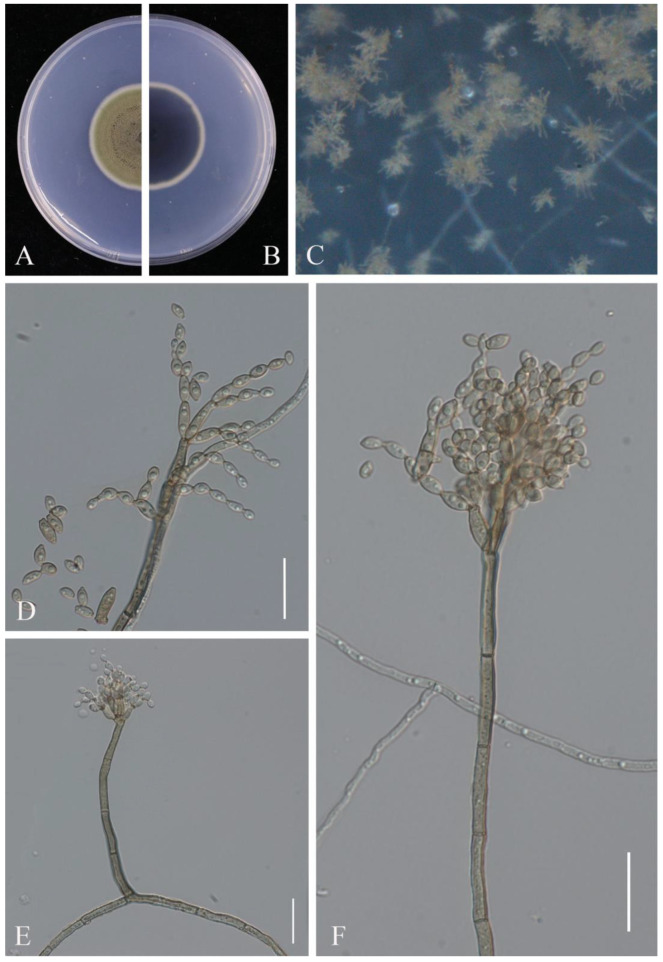
*Cladosporium guizhouense* (JZB390091): (**A**) upper view of the colony on PDA after seven days; (**B**) reverse view of the colony on PDA after seven days; (**C**) mycelia and conidiophore on SNA; (**D**–**F**) conidiophore, secondary ramoconidia, and conidia. Scale bars: (**D**–**F**) = 20 μm.

Notes: Two *Cladosporium* isolates generated in the present study (JZB390091, JZB390092) formed a sister clade with *C*. *guizhouense* strains with 100% ML bootstrap values and 1.0 Bayesian probabilities (Figure 6). Morphological characters of the two isolates (Figure 7) are similar to the original description of *C. guizhouense* [50]. *Cladosporium guizhouense* was introduced as a saprobe on fallen leaves of *Eucommia ulmoides* by Wang et al. [50] in Guizhou Province, China, and subsequently isolated from *Citrus reticulata* [51]. The species was also reported as a mycoparasite on rust fungi [52,53]. This is the first report of *C*. *guizhouense* on blueberry (*Vaccinium* spp.).

***Colletotrichum fioriniae*** (Moug.: Fr.) (Marcelino and Gouli) Pennycook, Mycotaxon 132(1): 150 (2017) [2016].

Index Fungorum: IF 553097; Facesoffungi Number: FoF 02891.

Classification: Glomerellaceae, Glomerellales, Hypocreomycetidae, Sordariomycetes, Ascomycota, Fungi [40].

*Pathogenic* on fruits of *Vaccinium* sp. For morphology, see description by Damm [54] (description and illustration) and Bell [6] (record on blueberry fruit).

Material examined: CHINA, Guizhou Province, isolated from diseased fruits of *Vaccinium* spp., May 2023, Y. Y. Zhou and X. H. Li (dry cultures JZBH330439, JZBH330440), living cultures JZB330439, JZB330440.

**Figure 8:**
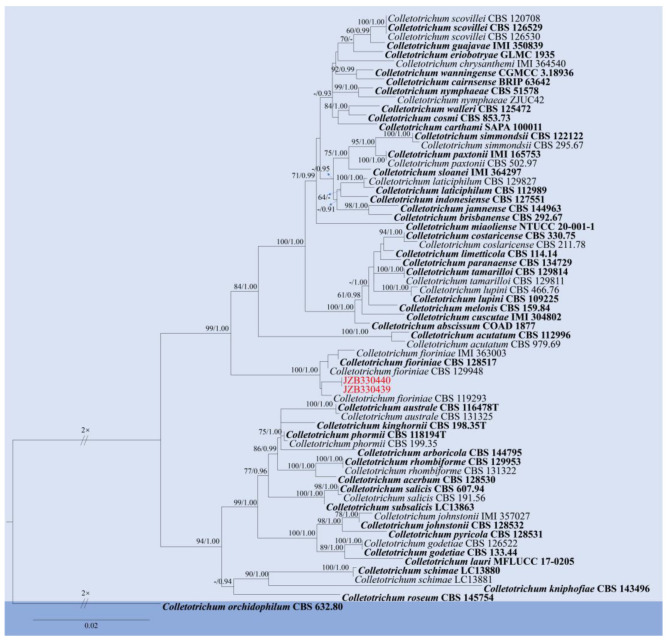
Phylogenetic tree generated by maximum likelihood (ML) analysis of combined ITS, *gapdh*, *chs*, and *act*, *β-tub* sequence data of *Colletotrichum acutatum* species complex. The tree is rooted with *Colletotrichum orchidophilum* (CBS 632.80). The matrix has 542 distinct alignment patterns, with 3.67% of characters or gaps undetermined. Estimated base frequencies are as follows: A = 0.231239, C = 0.296836; G = 0.244083; T = 0.227842. Substitution rates: AC = 1.611674; AG = 4.644984; AT = 1.380603; CG = 0.638683; CT = 7.444475; GT = 1.000000. Gamma distribution shape parameter: α = 0.920840. ML bootstrap support values ≥ 60% and Bayesian posterior probabilities (BYPP) ≥ 0.90 are given near the nodes. The scale bar indicates 0.02 changes. The arrowhead points toward the node to which the value belongs. The ex-type strains are in bold, and newly generated sequences are in red.

**Figure 9:**
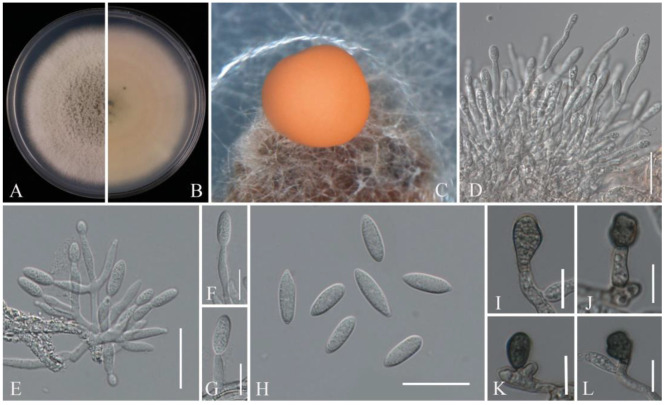
*Colletotrichum fioriniae* (JZB330439): (**A**) upper view of the colony on PDA after seven days; (**B**) reverse view of the colony on PDA after seven days; (**C**) conidial mass; (**D**–**G**) conidiophores; (**H**) conidia; (**I**–**L**) appressoria. Scale bars: (**D**,**E**,**H**) = 20 μm; (**F**,**G**,**I**–**L**) = 10 μm.

Notes: The two isolates obtained in this study (JZB330439, JZB330440) clustered with *C. fioriniae* strains in the phylogenetic analysis (100% ML bootstrap and 1.00 BYPP) (Figure 8) and share similar morphological characters (Figure 9) with the type-description of *C. fioriniae* [54]. *Colletotrichum fioriniae* is widely distributed around the world, especially in temperate regions [55]. This species is one of the most common *Colletotrichum* species in China, with more than 30 hosts, and has been isolated from the anthracnose fruit rot of litchi, peaches, and pears [28,56,57,58]. This is the first report of *C. fioriniae* causing fruit rot on blueberry (*Vaccinium* spp.) in China.

***Diaporthe anacardii*** (Early and Punith.) R.R. Gomes, Glienke and Crous, Persoonia 31: 15 (2013).

Index Fungorum: IF 802923; Facesoffungi Number: FoF 16978.

Classification: Diaporthaceae, Diaporthales, Diaporthomycetidae, Sordariomycetes, Ascomycota, Fungi [40].

*Pathogenic* on fruits of *Vaccinium* sp. **Sexual morph**: Not observed. **Asexual morph**: *Conidiomata*: pycnidial, globose, black. *Conidiophores*: 10–21.5 × 1–2 μm ($\bar{x}$ = 15.1 × 1.4 μm; n = 30), densely aggregated, branched, straight to slightly sinuous, smooth, hyaline, cylindrical, slightly tapering towards the apex. *Alpha conidia*: 6–9 × 2–3 μm ($\bar{x}\;$ = 7.6 × 2.8 μm; n = 50), straight, aseptate, bi-guttulate or multi-guttulate, hyaline, fusoid to ellipsoid, slightly tapering towards both ends. Beta conidia not observed.

Culture characteristics: Colonies on PDA reaching 78 mm diam. after five days at 25 °C, white with moderate, felted aerial mycelium, reverse yellowish to brownish at the center.

Material examined: CHINA, Fujian Province, isolated from diseased fruits of *Vaccinium* spp., May 2023, Y. Y. Zhou and X. H. Li (dry cultures JZBH320308), living cultures JZB320308.

**Figure 10:**
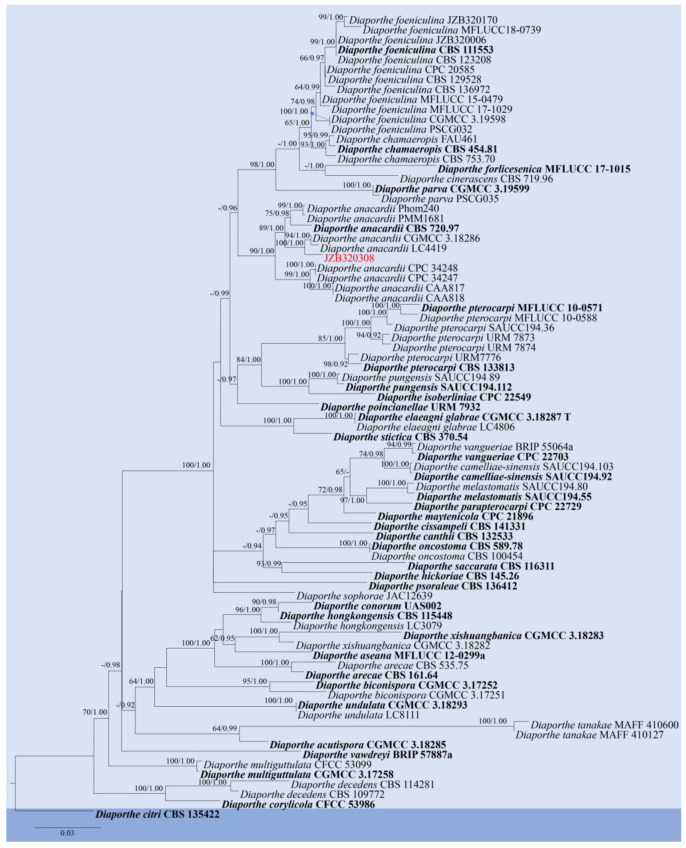
Phylogenetic tree generated by maximum likelihood (ML) analysis of combined ITS, *tef 1-α*, *β-tub*, *cal*, and *his* sequence data of Section Foeniculina of *Diaporthe* species. The tree is rooted with *Diaporthe citri* (CBS 135422). The matrix has 1121 distinct alignment patterns, with 25.45% of characters or gaps undetermined. Estimated base frequencies are as follows: A = 0.214637; C = 0.324631; G = 0.237836; T = 0.222897. Substitution rates: AC = 1.200371; AG = 3.247133; AT = 1.039432; CG = 0.794522; CT = 4.498676; GT = 1.000000. Gamma distribution shape parameter: α = 0.912183. ML bootstrap support values ≥ 60% and Bayesian posterior probabilities (BYPP) ≥ 0.90 are given near the nodes. The scale bar indicates 0.03 changes. The arrowhead points toward the node to which the value belongs. The ex-type strains are in bold, and newly generated sequences are in red.

**Figure 11:**
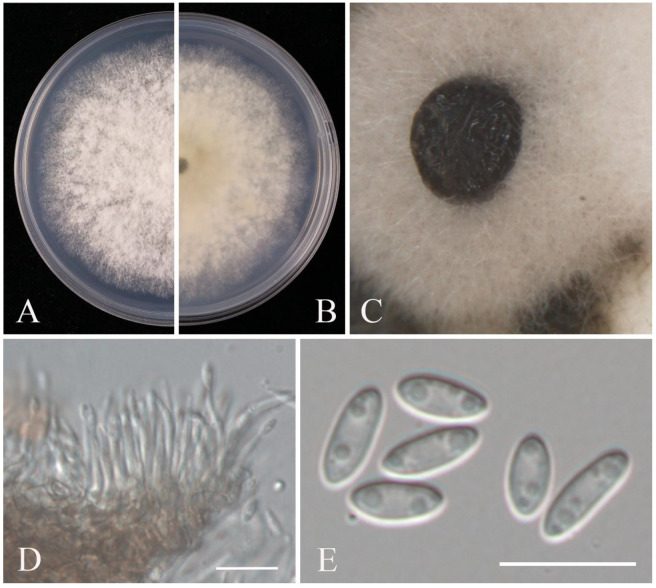
*Diaporthe anacardii* (JZB320308): (**A**) upper view of the colony on PDA after five days; (**B**) reverse view of the colony on PDA after three days; (**C**) conidiomata on PNA; (D) conidiophores; (**E**) alpha conidia. Scale bars: (**D**,**E**) = 10 μm.

Notes: For the taxonomic treatment of *Diaporthe*, we followed the latest classification proposed by Norphanphoun et al. [29] and Dissanayake et al. [59]. The phylogenetic analysis showed that our isolate, JZB320308, clustered within the clade of *Diaporthe anacardii*, sister to CGMCC 3.18286 and LC4419 (=*D. velutina*) (Figure 10), and the morphology characters of JZB320308 (Figure 11) conformed to the description of *D. anacardii* [60]. *Diaporthe anacardii* was introduced from *Anacardium occidentale* in Africa (as *Phomopsis anacardii*) [61] and was epitypified by Gomes et al. [62]. According to the new classification system, *D. acutispora*, *D. nebulae*, *D. phillipsii*, *D. portugallica*, and *D. velutina* are reduced to synonymy with *D. anacardii* [59]. Among them *D. phillipsii* was originally introduced as a pathogen associated with blueberry twig blight and dieback in Portugal [63]. This is the first report of *D. anacardii* causing fruit rot on blueberry in China.

***Fusarium annulatum*** Bugnic., Rev. gén. Bot. 59: 13 (1952).

Index Fungorum: IF 297536; Facesoffungi Number: FoF 16723.

Classification: Nectriaceae, Hypocreales, Hypocreomycetidae, Sordariomycetes, Ascomycota, Fungi [40].

*Pathogenic* on fruits of *Vaccinium* sp. **Sexual morph**: Not observed. **Asexual morph**: *Conidiophores* on CLA produced laterally on aerial mycelium and substrate mycelium, straight or flexuous, simple or more commonly sympodially to irregularly branched, or reduced to conidiogenous cells borne laterally on hyphae; *Conidiogenous cells*: mono- and polyphialidic, subulate to cylindrical. *Microconidia*: 5–10 × 2–3.5 μm ($\bar{x}\;$= 7.0 × 2.6 μm; n = 50), formed on aerial conidiophores, hyaline, obovoid to ellipsoidal, smooth- and thin-walled, aseptate, forming a false head on phialides. *Macroconidia*: 21.5–55.5 × 4–5.5 μm ($\bar{x}\;$= 40.9 × 4.5 μm; n = 30), sparse, straight to slightly curved, tapering toward the basal part; apical cell blunt; basal cell barely notched, 2–4-septate, hyaline, thin- and smooth-walled. *Chlamydospores* absent.

Culture characteristics: Colonies on PDA reaching 75 mm diam. after six days at 25 °C, flat, light purple to orange with abundant aerial mycelia.

Material examined: CHINA, Fujian Province, isolated from diseased fruits of *Vaccinium* spp., May 2023, Y. Y. Zhou and X. H. Li (dry cultures JZBH3110489, JZBH3110490), living cultures JZB3110489, JZB3110490.

**Figure 12:**
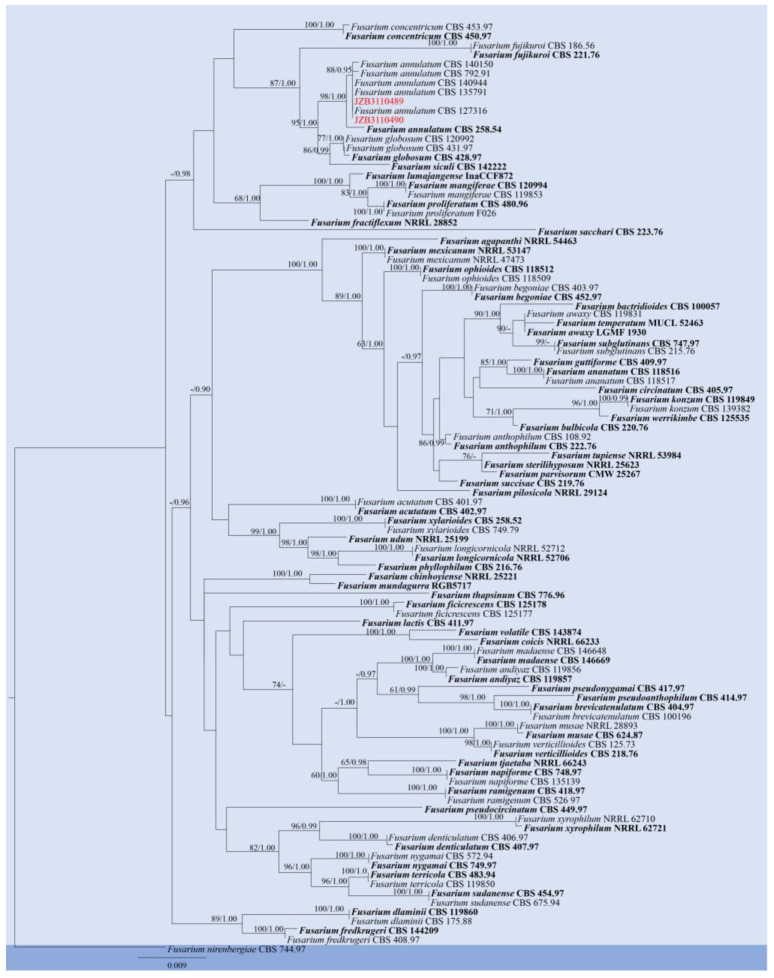
Phylogenetic tree generated by maximum likelihood (ML) analysis of combined *tef 1-α* and *rpb2* sequence data of *Fusarium* species. The tree is rooted with *Fusarium nirenbergiae* (CBS 744.97). The matrix has 421 distinct alignment patterns, with 3.70% of characters or gaps undetermined. Estimated base frequencies are as follows: A = 0.263854; C = 0.251445; G = 0.251382; T = 0.233319. Substitution rates: AC = 1.435916; AG = 6.155181; AT = 1.375277; CG = 0.854277; CT = 13.787190; GT = 1.000000. Gamma distribution shape parameter: α = 0.814503. ML bootstrap support values ≥ 60% and Bayesian posterior probabilities (BYPP) ≥ 0.90 are given near the nodes. The scale bar indicates 0.009 changes. The ex-type strains are in bold, and newly generated sequences are in red.

**Figure 13:**
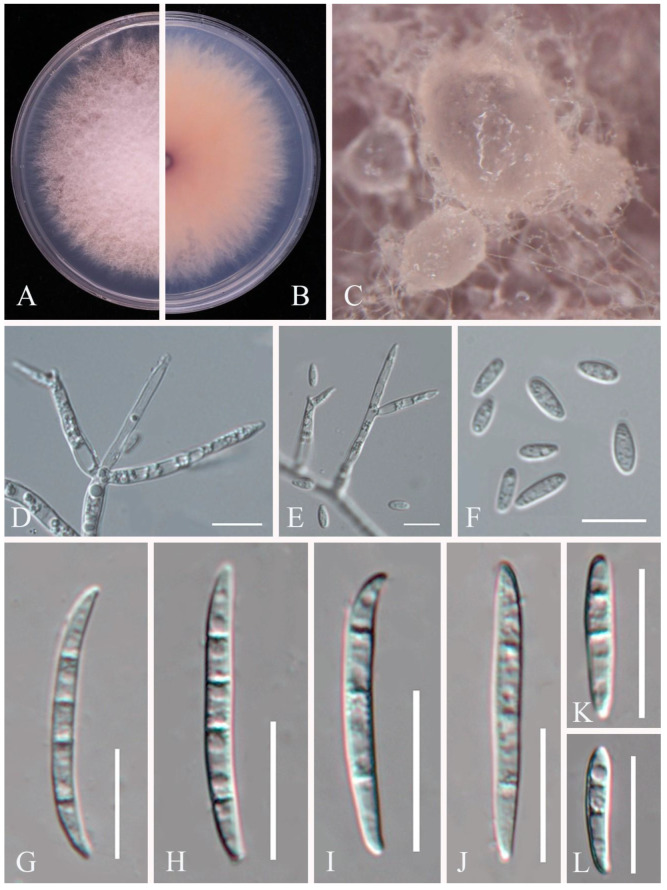
*Fusarium annulatum* (JZB3110489): (**A**) upper view of the colony on PDA after six days; (**B**) reverse view of the colony on PDA after six days; (**C**) sporodochia formed on CLA; (**D**,**E**) aerial conidiophores; (**F**) microconidia; (**G**–**L**) macroconidia. Scale bars: (**D**–**F**) = 10 μm; (**G**–**L**) = 20 μm.

Notes: According to the phylogenetic analyses, two isolates in this study (JZB3110489, JZB3110490) clustered within the clade of *Fusarium annulatum* (Figure 12). *Fusarium annulatum* was introduced by Bugnicourt [64] from *Oryza sativa* and was reported to infect multiple plant hosts under the name *F. proliferatum* [65]. Yilmaz et al. [30] fixed the typification of *F. proliferatum* to a distinct phylogenetic clade and provided an emended description of *F. annulatum*. *Fusarium annulatum* showed phylogenetic diversity and differentiation in morphology. According to the original description of the type-strain (CBS 258.54) by Bugnicourt [64], sporodochial conidia of *F. annulatum* are strongly curved and almost ring-shaped, and Nelson et al. [66] regraded *F. annulatum* as *F. proliferatum* with strongly curved, sporodochial conidia (macroconidia), while Yilmaz et al. [30] found that most *F. annulatum* isolates only produce straight, sporodochial conidia. Our study confirmed that macroconidia are straight to slightly curved (Figure 13). *Fusarium annulatum* was reported to cause blight on *Bletilla striata*, maize, and *Rosa roxburghii* rot disease in China [67,68,69]. This is the first report of *F. annulatum* causing fruit rot on blueberry (*Vaccinium* spp.).

***Neopestalotiopsis surinamensis*** Maharachch., K.D. Hyde and Crous, in Maharachchikumbura, Hyde, Groenewald, Xu and Crous, Stud. Mycol. 79: 149 (2014).

Index Fungorum: IF 809781; Facesoffungi Number: FoF 16979.

Classification: Sporocadaceae, Amphisphaeriales, Xylariomycetidae, Sordariomycetes, Ascomycota, Fungi [40].

*Pathogenic* on fruits of *Vaccinium* sp. **Sexual morph**: Not observed. **Asexual morph**: *Conidiomata*: subglobose to globose, scattered or gregarious, semi-immersed or erumpent, exuding globose, black conidial masses. *Conidiophores*: indistinct, often reduced to conidiogenous cells. *Conidiogenous cells*: discrete, ampulliform, flask-shaped to subcylindrical, smooth-walled, hyaline. *Conidia*: 20–27 × 7–9.5 μm ($\bar{x}\;$ = 23.0 × 8.1 μm; n = 30), fusoid, ellipsoid to subcylindrical, straight to slightly curved, 4-septate; basal cell 3.5–5 μm long, obconic with a truncate base, hyaline, rugose and thin-walled; three median cells 14–18.5 μm ($\bar{x}\;$ = 15.7 μm; n = 30) long, doliiform, wall rugose, versicolored, brown to pale brown, septa darker than the rest of the cell, second cell from the base 5–6 μm long, pale brown to brown; third cell 4.5–6 μm long, brown; fourth cell 4.5–6 μm long, brown; apical cell 3–4 μm long, hyaline, conical to subcylindrical, thin- and smooth-walled; apical appendages 2–4, 12.5–28.5 μm ($\bar{x}\;$ = 20.2 μm; n = 30) long, tubular, arising from the apical crest, unbranched, filiform; basal appendage 3.5–7.5 μm ($\bar{x}\;$ = 5.0 μm; n = 30) long, single, tubular, unbranched, centric.

Culture characteristics: Colonies on PDA reaching 79 mm diam. after five days at 25 °C, raised with lobate margin, white, reverse pale yellow, aerial mycelia dense, flocculent.

Material examined: CHINA, Fujian Province, isolated from diseased fruits of *Vaccinium* spp., May 2023, Y. Y. Zhou and X. H. Li (dry cultures JZBH340093–JZBH340095), living cultures JZB340093–JZB340095.

**Figure 14:**
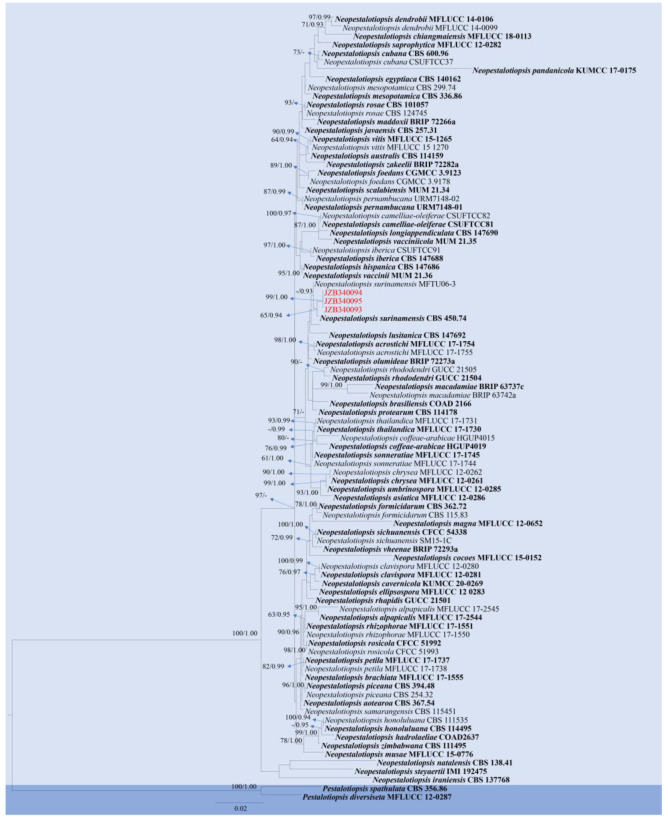
Phylogenetic tree generated by maximum likelihood (ML) analysis of combined ITS, *tef 1-α*, and *rpb2* sequence data of *Neopestalotiopsis* species. The tree is rooted with *Pestalotiopsis spathulate* (CBS 356.86) and *Pestalotiopsis diversiseta* (MFLUCC 12-0287). The matrix has 606 distinct alignment patterns, with 13.33% of characters or gaps undetermined. Estimated base frequencies are as follows: A = 0.234657; C = 0.273247; G = 0.214726; T = 0.277370. Substitution rates: AC = 1.150331; AG = 1.150331; AT = 1.150331; CG = 0.850459; CT = 4.628454; GT = 1.000000. Gamma distribution shape parameter: α = 0.898468. ML bootstrap support values ≥ 60% and Bayesian posterior probabilities (BYPP) ≥ 0.90 are given near the nodes. The scale bar indicates 0.009 changes. The arrowhead points toward the node to which the value belongs. The ex-type strains are in bold, and newly generated sequences are in red.

**Figure 15:**
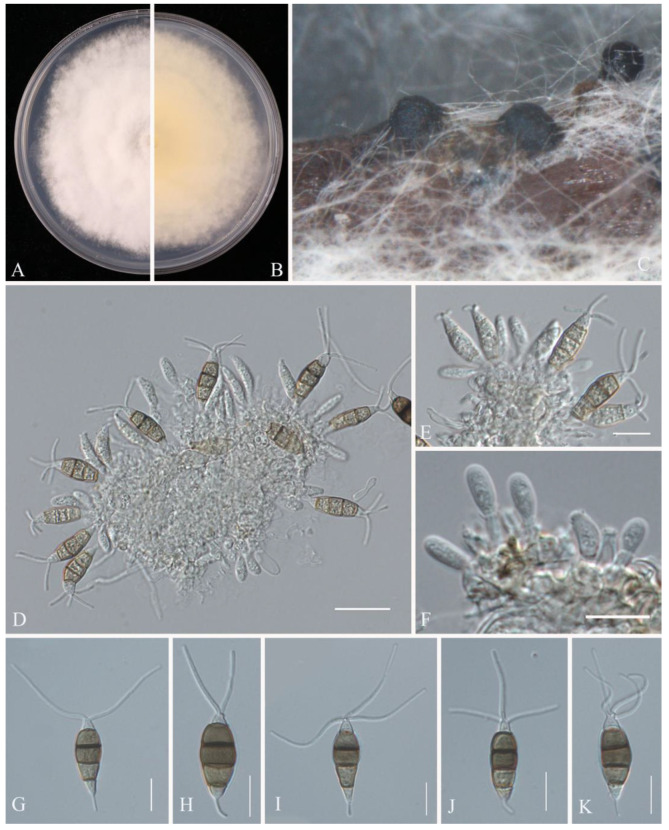
*Neopestalotiopsis surinamensis* (JZB340093): (**A**) upper view of the colony on PDA after five days; (**B**) reverse view of the colony on PDA after five days; (**C**) conidiomata on PNA; (**D**–**F**) conidiogenous cells; (**G**–**K**) conidia. Scale bars: (**D**) = 20 μm; (**E**–**K**) = 10 μm.

Notes: In the phylogenetic tree of *Neopestalotiopsis*, three isolates from this study (JZB340093–JZB340095) grouped with the ex-type strain of *Neopestalotiopsis surinamensis* (CBS 450.74) and another strain (MFTU06-3) (Figure 14). Morphologically, isolates in this study (Figure 15) were similar to the original description of *N. surinamensis* by Maharachchikumbura et al. [70]. However, minor dimensional differences were observed in the size of the basal cell and the apical cell, which may be attributed to host differences. *Neopestalotiopsis surinamensis* was introduced from the soil under *Elaeis guineensis* in *Suriname* and living leaves of *Protea eximia* in Zimbabwe [70] and was reported as a pathogen causing guava scab [71]. This is the first report of *Neopestalotiopsis surinamensis* causing blueberry fruit rot.

### 3.3. Pathogenicity Test

**Figure 16:**
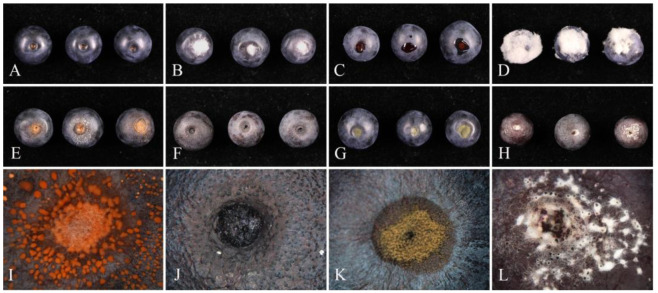
Detached fruit pathogenicity test for the fungal isolates on blueberry fruits five days post inoculation: (**A**) control (sterilized water); (**B**) *Fusarium annulatum* (JZB3110489); (**C**) *Diaporthe anacardii* (JZB320308); (**D**) *Botrytis cinerea* (JZB350048); (**E**,**I**) *Colletotrichum fioriniae* (JZB330439); (**F**,**J**) *Botryosphaeria dothidea* (JZB310278); (**G**,**K**) *Cladosporium guizhouense* (JZB390091); (**H**,**L**) *Neopestalotiopsis surinamensis* (JZB340093).

Among the sixteen isolates obtained from blueberry fruit rot, one representative isolate from each genus was selected for pathogenicity assay. These include *Botryosphaeria dothidea* (JZB310278), *Botrytis cinerea* (JZB350048), *Cladosporium guizhouense* (JZB390091), *Colletotrichum fioriniae* (JZB330439), *Diaporthe anacardii* (JZB320308), *Fusarium annulatum* (JZB3110489), and *Neopestalotiopsis surinamensis* (JZB340093). All experimental isolates showed virulence on blueberry fruits with different symptoms (Figure 16). Fruits inoculated with *F*. *annulatum* formed white mycelia at the inoculation site; fruits inoculated with *D*. *anacardii* showed soft rot with a fluid exuded from the inoculation site; fruits inoculated with *B*. *cinerea* showed a rot surrounded by grey white mycelia; fruits inoculated with *C*. *fioriniae* secreted orange conidial mass; fruits inoculated with *B*. *dothidea* shrivelled and produced black globose conidiomata; fruits inoculated with *C*. *guizhouense* produced lesion with olive-green colony, and fruits inoculated with *N*. *surinamensis* produced a lesion shrivelled and formed white mycelia and black conidiomata. No symptoms were induced in the control fruits inoculated with sterile water. Fungal pathogens were re-isolated from lesions and confirmed by the morphology and ITS sequence to prove Koch’s postulates.

## 4. Discussion

In this study, fungal species associated with blueberry fruit rot were identified by morphological characterization and phylogenetic analyses. Among the seven species identified, *Botrytis cinerea* and *Colletotrichum fioriniae* have previously been recognized as important pathogens of blueberry fruit rot [4]. Grey mould caused by *Botrytis cinerea* could occur in open fields and greenhouses, affecting blueberries from the seedling stage to post-harvest. Rainfall and high humidity during the flowering period of blueberry were more conducive to the occurrence of severe grey mould [72]. In China, Dai et al. [73] isolated *B. cinerea* from blueberry fruits, identified by morphology. The current study confirmed the species by morphology and phylogeny. For *Colletotrichum fioriniae*, the pathogenicity on blueberry fruit was initially conducted by MacKenzie et al. [74] in Florida, USA, while the test strains were regarded as *C. acutatum*. Subsequently, Damm et al. [54] accommodated those strains into *C. fioriniae*. Eaton et al. [10] identified the *Colletotrichum* species causing fruit rots in mixed-fruit orchards in Kentucky, USA, and found that all isolates from blueberry in the study belonged to *C. fioriniae*. In recent years, blueberry fruit disease caused by *C. fioriniae* has also been reported in Chile, Mexico, New Zealand, and Poland [75,76,77], and the species is first reported in China in the current study. *Botryosphaeria dothidea* is usually reported as the causal agent of blueberry stem blight, together with other Botryosphaeriaceae species [78]. Zhang et al. [44] investigated fruit rot in Guizhou Province in China, and the symptoms include rot and necrosis from the pedicel, with raised black spots on the fruit surface. The pathogen was identified as *B. dothidea* by morphology. In this study, *B. dothidea* from fruit rot in Fujian Province was determined by morphology and phylogenetic analysis. *Diaporthe* is well-known as a genus comprising plant pathogens, endophytes, or saprobes, with a wide distribution and a range of hosts [29,62,79]. On blueberry, the genus has been reported to cause stem blight and fruit rot or to occur as endophytes or a latent pathogen [63]. *Diaporthe vaccinii* can infect blueberry fruits throughout the entire growth period but often remains latent until the mature stages/harvest period [80]. Furthermore, *D. nobilis* was reported to cause post-harvest rot of blueberry. Nevertheless, the two species were all synonymized under *D. eres* [81]. The isolates generated in this study clustered with *D. velutina*, which has been synonymized under *D. anacardii* [59]. Among the species reduced to synonymy with *D. anacardii*, *D. phillipsii* was introduced from blueberry, associated with stem dieback. According to the two studies, current *D. anacardii* could be a prevalent pathogen in blueberry.

*Neopestalotiopsis* is one of the major causal agents of twig dieback, stem blight, and canker, as well as leaf spot on blueberry [82], while there are rare records of *Neopestalotiopsis* on blueberry fruit diseases. However, *Neopestalotiopsis* spp. have been reported to cause fruit rots on various hosts such as strawberry, plum, and avocado [83]. *Fusarium* is a common pathogen on blueberry, causing wilt and root rot [84,85], and *F. acuminatum* was reported to cause post-harvest fruit rot [86]. *Cladosporium* is a cosmopolitan genus comprising phytopathogens, saprobes, endophytes, and human pathogens [87,88]. As fruit pathogens, *Cladosporium* spp. were reported on dekopon, grapevine, and raspberry [89,90,91], and *Cladosporium cladosporioides* has been reported to cause blueberry fruit rot [77]. Our species from the above three genera, i.e., *Cladosporium guizhouense*, *Fusarium annulatum*, and *Neopestalotiopsis surinamensis*, are new host records on blueberry.

The pathogenicity of the seven species on blueberry was confirmed by the appearance of distinct and commonly reported symptoms. Fruits inoculated with the *Botrytis cinerea* and *Colletotrichum fioriniae* exhibited typical symptoms of grey mould and anthracnose, respectively [4,11]. The pathogenicity test of *Botryosphaeria dothidea* validated the fruit rot symptoms, presenting sunken lesion with black spots described by Zhang et al. [44]. *Diaporthe anacardii* caused fruit soft rot with juice oozing, resembling the symptoms caused by *D. eres* [80]. The newly collected isolates indicate the re-emergence of these important pathogens on blueberries. Although *Cladosporium guizhouense*, *Fusarium annulatum*, and *Neopestalotiopsis surinamensis* are reported on blueberries for the first time, the isolates obtained in this study showed strong virulence, producing typical symptoms associated with their respective genera. Given the significant role of these genera among plant pathogens, these species could be prevalent or exist as latent pathogens on blueberries. As many pathogens in the past were identified solely based on morphological characters, it is likely that these pathogens had already existed but remained undetected due to the limitations of traditional identification methods.

Though many studies have highlighted post-harvest management, many post-harvest diseases originate from infections in the field [92]. Infections start at bloom but can remain latent until fruit ripening or express symptoms during the post-harvest period [4]. Latent pathogens can be activated by favorable conditions, environmental stresses, and mechanical injuries [4]. Diseased fruits may be harvested together with healthy fruits in a cluster of blueberries and cause persistent infection by contaminating healthy fruit, resulting in significant losses [4,13]. Thus, preharvest management, such as disease monitoring and agricultural and chemical control, is crucial for protecting fruit health [4,92].

In conclusion, this study comprehensively and accurately identified the fungal pathogens responsible for blueberry fruit rot disease, demonstrating that the disease in the field is caused by a mixed infection of multiple genera and species rather than a single pathogen. The results enhance the understanding of fruit rot disease occurrence and provide a valuable reference for the development of prevention and control strategies, such as the selection of the targeted fungicide and establishing effective treatment schedules. Since disease severity is affected by pathogens, climate factors, and blueberry variety, further studies are necessary to explore the occurrence regularity of diseases, pathogen regional distribution, and virulence differences among host cultivars.

## Figures and Tables

**Table 1:** Primers used in the present study with sequences and annealing temperature.

Locus	Primer	Sequence of Primer (5′–3′)	Annealing Temperature (°C)	Reference
*act*	ACT-512F	ATGTGCAAGGCCGGTTTCGC	59	[14]
	ACT-783R	TACGAGTCCTTCTGGCCCAT		
ITS	ITS5	GGAAGTAAAAGTCGTAACAAGG	58	[15]
	ITS4	TCCTCCGCTTATTGATATGC		
*cal*	CL1C	GAATTCAAGGAGGCCTTCTC	59	[16]
	CL2C	CTTCTGCATCATGAGCTGGAC		
	CAL-228F	GAGTTCAAGGAGGCCTTCTCCC	54	[14]
	CAL-737R	CATCTTTCTGGCCATCATGG		
*chs*	CHS-79F	TGGGGCAAGGATGCTTGGAAGAAG	58	[14]
	CHS-345R	TGGAAGAACCATCTGTGAGAGTTG		
*gapdh*	G3PDHfor	ATTGACATCGTCGCTGTCAACGA	64	[17]
	G3PDHrev	ACCCCACTCGTTGTCGTACCA		
	GDF	GCCGTCAACGACCCCTTCATTGA	59	[18]
	GDR	GGGTGGAGTCGTACTTGAGCATGT		
*his*	CYLH3F	AGGTCCACTGGTGGCAAG	58	[19]
	H3-1b	GCGGGCGAGCTGGATGTCCTT		
*hsp60*	HSP60for	CAACAATTGAGATTTGCCCACAAG	55	[17]
	HSP60rev	GATGGATCCAGTGGTACCGAGCAT		
*rpb2*	rpb2-5f2	GGGGWGAYCAGAAGAAGGC	56	[20]
	rpb2-7cR	CCCATRGCTTGYTTRCCCAT		[21]
	RPB2Ffor	GATGATCGTGATCATTTCGG	55	[17]
	RB2rev	CCCATAGCTTGCTTACCCAT		
*tef 1-α*	EF1-728F	CATCGAGAAGTTCGAGAAGG	54	[14]
	EF1-986R	TACTTGAAGGAACCCTTACC		
	EF1-688F	CGGTCACTTGATCTACAAGTGC	54	[22]
	EF1-1251R	CCTCGAACTCACCAGTACCG		
	EF1	ATGGGTAAGGARGACAAGAC	55	[23]
	EF2	GGARGTACCAGTSATCATG		
*β-tub*	T1	AACATGCGTGAGATTGTAAGT	58	[24]
	Bt2a	GGTAACCAAATCGGTGCTGCTTTC	58	[19]
	Bt2b	ACCCTCAGTGTAGTGACCCTTGGC		

**Table 2:** Primers for amplification of gene loci of each fungal genus in the study.

Genus	Loci Used for Amplification										Reference
	ITS	*β-tub*	*tef 1-α*	*gapdh*	*cal*	*act*	*chs*	*rpb2*	*his*	*hsp60*	
*Botryosphaeria*	ITS5/ ITS4	Bt2a/ Bt2b	EF1-728F/ EF1-986R								[25]
*Botrytis*				G3PDHfor/ G3PDHrev				RPB2for/RPB2rev		HSP60for/HSP60rev	[26]
*Cladosporium*	ITS5/ ITS4		EF1-728F/ EF1-986R			ACT-512F/ ACT-783R					[27]
*Colletotrichum*	ITS5/ ITS4	T1/Bt2b		GDF/ GDR		ACT-512F/ ACT-783R	CHS-79F/ CHS-345R				[28]
*Diaporthe*	ITS5/ ITS4	Bt2a/ Bt2b	EF1-688F/ EF1-1251R		CAL-228F/ CAL-737R				CYLH3F/ H31b		[29]
*Fusarium*			EF1/EF2					RPB2-5f2/ 7cR			[30]
*Pestalotiopsis*	ITS5/ ITS4	T1/Bt2b	EF1-688F/ EF1-1251R								[31]

## Data Availability

All the sequence data generated in this study are available in GenBank (https://www.ncbi.nlm.nih.gov/nucleotide/), and the accession numbers are stated in the article.

## Supplementary Materials

The following supporting information can be downloaded at https://www.mdpi.com/article/10.3390/pathogens14020201/s1: Table S1: GenBank accession numbers of the isolates generated in the present study.
