# Supplementary material for: Characterization of Fungal Pathogens Causing Blueberry Fruit Rot Disease in China
Source: Pathogens. 2025 Feb 18;14(2):201. doi: 10.3390/pathogens14020201 (PMC11858039; doi:10.3390/pathogens14020201)
Supplement: Supplementary file 1 [file pathogens-14-00201-s001.zip › Supplementary.pdf]

Supplementary

Table S1. GenBank accession numbers of the isolates generated in the present study

**Species**	**Previous species name**	**Culture collection number**	**ITS**	***tef***	***tub***		
Botryosphaeria agaves		CBS 133992 [T]	JX646791	JX646856	JX646841		
Botryosphaeria agaves		MFLUCC 10-0051	JX646790	JX646855	JX646840		
Botryosphaeria corticis		CBS 119047 [T]	DQ299245	EU017539	EU673107		
Botryosphaeria corticis		ATCC 22927	DQ299247	EU673291	EU673108		
Botryosphaeria dothidea		CBS 115476 [T]	AY236949	AY236898	AY236927		
Botryosphaeria dothidea		CBS 110302	AY259092	AY573218	EU673106		
Botryosphaeria dothidea	*Botryosphaeria auasmontanum*	CBS 121769	EU101303	EU101348	N/A		
Botryosphaeria dothidea	*Botryosphaeria minutispermati a*	GZCC 16-0013	KX447675	KX447678	N/A		
Botryosphaeria dothidea	*Botryosphaeria sinensis*	CGMCC3.17723	KT343254	KU221233	KX197107		
Botryosphaeria dothidea	*Botryosphaeria wangensis*	CERC2298	KX278002	KX278107	KX278211		
Botryosphaeria dothidea		**JZB310277**	**PQ569347**	**PQ573068**	**PQ573070**		
Botryosphaeria dothidea		**JZB310278**	**PQ569348**	**PQ573069**	**PQ573071**		
Botryosphaeria fabicerciana		CBS 127193 [T]	HQ332197	HQ332213	KF779068		
Botryosphaeria fabicerciana		CBS 127194	HQ332198	HQ332214	KF779069		
Botryosphaeria kuwatsukai		CBS 135219 [T]	KJ433388	KJ433410	N/A		

Botryosphaeria kuwatsukai		LSP 5	KJ433395	KJ433417	N/A		
Botryosphaeria puerensis		CGMCC 3.2008 T	MT028569	MT028735	MT028901		
Botryosphaeria qingyuanensis		CERC2946 T	KX278000	KX278105	KX278209		
Botryosphaeria qingyuanensis		CERC2947	KX278001	KX278106	KX278210		
Botryosphaeria ramosa		CBS 122069 T	EU144055	EU144070	KF766132		
Botryosphaeria ramosa		CERC 2001	KX277989	KX278094	KX278198		
Botryosphaeria scharifii		CBS 124703 T	JQ772020	JQ772057	N/A		
Botryosphaeria scharifii		CBS 124702	JQ772019	JQ772056	N/A		
Cophinforma eucalypti		CBS 134651 T	JX646800	JX646865	JX646848		
**Species**	**Previous species name**	**Culture collection number**	***rpb2***	***gapdh***	***his***		
Botrytis aclada		MUCL8415	AJ745664	AJ704992	AJ716050		
Botrytis aclada		PRI006	AJ745665	AJ704993	AJ716051		
Botrytis allii		MUCL403	AJ745666	AJ704996	AJ716055		
Botrytis byssoidea		MUCL94 T	AJ745670	AJ704998	AJ716059		
Botrytis californica		X655 T	KJ937049	KJ937069	KJ937059		
Botrytis californica		X503	KJ937048	KJ937068	KJ937058		
Botrytis calthae		MUCL1089	AJ745672	AJ705000	AJ716061		
Botrytis calthae		MUCL2830	AJ745673	AJ705001	AJ716062		
Botrytis caroliniana		CB15 T	JF811590	JF811584	JF811587		
Botrytis caroliniana		WM4	JF811591	JF811585	JF811588		
Botrytis caroliniana		CA3	JF811592	JF811586	JF811589		

Botrytis cinerea		MUCL87 [T]	AJ745676	AJ705004	AJ716065		
Botrytis cinerea		Bc21	AM231317	AM231158	AM232675		
Botrytis cinerea		**JZB350048**	**PQ573080**	**PQ573072**	**PQ573076**		
Botrytis cinerea		**JZB350049**	**PQ573081**	**PQ573073**	**PQ573077**		
Botrytis cinerea		**JZB350050**	**PQ573082**	**PQ573074**	**PQ573078**		
Botrytis cinerea		**JZB350051**	**PQ573083**	**PQ573075**	**PQ573079**		
Botrytis convoluta		MUCL11595	AJ745680	AJ705008	AJ716069		
Botrytis convoluta		9801	AJ745679	AJ705007	AJ716068		
Botrytis croci		MUCL436	AJ745681	AJ705009	AJ716070		
Botrytis deweyae		CBS 134649 [T]	HG799518	HG799521	HG799519		
Botrytis elliptica		BE9714	AJ745684	AJ705012	AJ716073		
Botrytis elliptica		Be9605	AM231318	AM231168	AM232668		
Botrytis eucalypti		CERC 7170 [T]	KX301028	KX301020	KX301024		
Botrytis eucalypti		CERC 7208	KX301029	KX301021	KX301025		
Botrytis euroamericana		CBS 141699 [T]	KC191679	KC191677	KC191678		
Botrytis euroamericana		HA06	KX266740	KX266728	KX266734		
Botrytis fabae		MUCL98 [T]	AJ745686	AJ705014	AJ716075		
Botrytis fabae		CBS 109.57	AJ745685	AJ705013	AJ716074		
Botrytis fabiopsis		BC-2 [T]	EU514473	EU519211	EU514482		
Botrytis fabiopsis		BC-13	EU563115	EU563109	EU563100		
Botrytis ficariarum		CBS 176.73 [T]	AJ745687	AJ705015	AJ716076		
Botrytis ficariarum		MUCL376	AJ745688	AJ705016	AJ716077		
Botrytis fragariae		D13_F_Me3	KX429710	KX429703	KX429696		
Botrytis fragariae		D13_H_J2-34	KX429712	KX429705	KX429698		
Botrytis galanthina		MUCL435	AJ745689	AJ705018	AJ716079		

Botrytis galanthina		MUCL3204	AJ745690	AJ705017	AJ716078		
Botrytis gladiolorum		9701	AJ745691	AJ705019	AJ716080		
Botrytis gladiolorum		MUCL3865	AJ745692	AJ705020	AJ716081		
Botrytis globosa		MUCL21514	AJ745694	AJ705021	AJ716082		
Botrytis globosa		MUCL444	AJ745693	AJ705022	AJ716083		
Botrytis hyacinthi		MUCL442	AJ745696	AJ705024	AJ716085		
Botrytis hyacinthi		0001	AJ745695	AJ705023	AJ716084		
Botrytis macadamiae		BRIP 72276a	MZ356232	MZ344225	MZ344236		
Botrytis macadamiae		BRIP 72295a [T]	MZ356233	MZ344226	MZ344237		
Botrytis mali		BPI412756 [T]		EF367129			
Botrytis medusae		B-555 [T]	MH732870	MH732861	MH732866		
Botrytis narcissicola		MUCL2120	AJ745697	AJ705026	AJ716087		
Botrytis narcissicola		MUCL18857	AJ745698	AJ705025	AJ716086		
Botrytis paeoniae		MUCL16084	AJ745700	AJ705028	AJ716089		
Botrytis paeoniae		0003	AJ745699	AJ705027	AJ716088		
Botrytis pelargonii		CBS 497.50 [T]	AJ745662	AJ704990	AJ716046		
Botrytis pelargonii		MUCL1152	AJ745701	AJ705029	AJ716090		
Botrytis polyblastis		CBS 287.38 [T]	AJ745702	AJ705030	AJ716091		
Botrytis polyphyllae		YN1	MG431975	MG387957	MG431978		
Botrytis polyphyllae		YN2	MG431976	MG387958	MG431979		
Botrytis polyphyllae		CGMCC 3.18842 [T]	MG431977	MG387959	MG431980		
Botrytis porri		MUCL3234 [T]	AJ745704	AJ705032	AJ716093		
Botrytis porri		MUCL3349	AJ745705	AJ705033	AJ716094		
Botrytis prunorum		Bpru-8	KP339986	KP339979	KP339993		

Botrytis prunorum		Bpru-21 [T]	KP339987	KP339980	KP339994		
Botrytis pseudocinerea		Bp-362	MH732871	MH732860	MH732865		
Botrytis pseudocinerea		ICMP19668		KC620370	KC620323		
Botrytis pyriformis		SedsarBC-1	KJ543492	KJ543484	KJ543488		
Botrytis pyriformis		SedsarBC-2	KJ543493	KJ543485	KJ543489		
Botrytis ranunculi		CBS 178.63 [T]	AJ745706	AJ705034	AJ716095		
Botrytis sinoallii		OnionBC-23 [T]	EU514479	EU519217	EU514488		
Botrytis sinoviticola		GBC-3-2b [T]	JN692427	JN692413	JN692399		
Botrytis sinoviticola		GBC-3-1c	JN692424	JN692410	JN692396		
Botrytis sphaerosperma		MUCL21481	AJ745708	AJ705035	AJ716096		
Botrytis sphaerosperma		MUCL21482	AJ745709	AJ705036	AJ716097		
Botrytis squamosa		MUCL1107 [T]	AJ745710	AJ705037	AJ716098		
Botrytis squamosa		MUCL9112	AJ745711	AJ705038	AJ716099		
Botrytis tulipae		BT9830	AJ745713	AJ705041	AJ716102		
Botrytis tulipae		Bt9806	AM231328	AM231175	AM232682		
Sclerotinia sclerotiorum		484	AJ745716	AJ705044	AJ716048		
**Species**	**Previous species name**	**Culture collection number**	**ITS**	***act***	***tef***		
Cladosporium acalyphae		CBS 125982 [T]	HM147994	HM148481	HM148235		
Cladosporium alboflavescens		CBS 140690 [T]	LN834420	LN834604	LN834516		
Cladosporium angulosum		CBS 140692 [T]	LN834425	LN834609	LN834521		
Cladosporium angulosum		COAD 2500	MK253346	MK249989	MK293786		
Cladosporium		CBS 125983 [T]	HM147995	HM148482	HM148236		

angustisporum							
Cladosporium angustiterminale		CBS 140480 T	KT600379	KT600575	KT600476		
Cladosporium anthropophilum		CBS 140685 T	LN834437	LN834621	LN834533		
Cladosporium anthropophilum		CPC 22393	MF472922	MF473772	MF473349		
Cladosporium arenosum		CHFC-EA 566 T	MN879328	MN890008	MN890011		
Cladosporium asperulatum		CBS 126340 T	HM147998	HM148485	HM148239		
Cladosporium australiense		CBS 125984 T	NR_119837	HM148486	HM148240		
Cladosporium austroafricanum		CBS 140481 T	KT600381	KT600577	KT600478		
Cladosporium austrolitorale		CBS 148321 T	MN879327	MN890007	MN890010		
Cladosporium caprifimosum		FMR 16532 T	LR813198	LR813205	LR813210		
Cladosporium cavernicola		URM 8389 T	MZ518829	MZ555746	MZ555733		
Cladosporium chalastosporoides		CBS 125985 T	HM148001	HM148488	HM148242		
Cladosporium chasmanthicola		CPC 21300 T	NR_152307	KY646224	KY646227		
Cladosporium chubutense		CBS 124457 T	FJ936158	FJ936165	FJ936161		
Cladosporium cladosporioides		CBS 112388 T	NR_119839	HM148490	HM148244		
Cladosporium cladosporioides		CBS 113738	HM148004	HM148491	HM148245		

Cladosporium colocasiae		CBS 386.64 T	HM148067	HM148555	HM148310		
Cladosporium colocasiae		CBS 119542	HM148066	HM148554	HM148309		
Cladosporium colombiae		CBS 274.80B T	FJ936159	FJ936166	FJ936163		
Cladosporium coprophilum		FMR 16164 T	LR813201	LR813207	LR813213		
Cladosporium crousii		CBS 140686 T	LN834431	LN834615	LN834527		
Cladosporium cucumerinum		CBS 171.52 T	NR_119841	HM148561	HM148316		
Cladosporium cucumerinum		CBS 176.54	HM148078	HM148567	HM148322		
Cladosporium delicatulum		CBS 126344 T	HM148081	HM148570	HM148325		
Cladosporium devikae		BRIP 72278a T	MZ303808	MZ344212	MZ344193		
Cladosporium endoviticola		JZB390018 T		MN984220	MN984228		
Cladosporium endoviticola		JZB390019		MN984221	MN984229		
Cladosporium eucommiae		GUCC 401.1 T	OL587465	OL519775	OL504966		
Cladosporium europaeum		CBS 134914 T	HM148056	HM148543	HM148298		
Cladosporium europaeum		FP-027-A9	MH102078	MH102068	MH102121		
Cladosporium exasperatum		CBS 125986 T	HM148090	HM148579	HM148334		
Cladosporium exile		CBS 125987 T	HM148091	HM148580	HM148335		
Cladosporium flabelliforme		CBS 126345 T	HM148092	HM148581	HM148336		
Cladosporium flavovirens		CBS 140462 T	LN834440	LN834624	LN834536		
Cladosporium funiculosum		CBS 122129 T	NR_119845	HM148583	HM148338		
Cladosporium funiculosum		CBS 122128	HM148093	HM148582	HM148337		
Cladosporium fuscoviride		FMR 16385	LR813200	LR813206	LR813212		
Cladosporium gamsianum		CBS 125989 T	HM148095	HM148584	HM148339		
Cladosporium globisporum		CBS 812.96 T	HM148096	HM148585	HM148340		

Cladosporium grevilleae		CBS 114271 [T]	JF770450	JF770473	JF770472		
Cladosporium guizhouense		GUCC 401.7 [T]	OL579741	OL519780	OL504965		
Cladosporium guizhouense		GUCC 401.8	ON334728	ON383338	ON383470		
Cladosporium guizhouense		COAD 3471	OP535382	OP598134	OP676093		
Cladosporium guizhouense		**JZB390091**	**PQ569370**	**PQ573084**	**PQ573086**		
Cladosporium guizhouense		**JZB390092**	**PQ569371**	**PQ573085**	**PQ573087**		
Cladosporium hemileiicola		COAD 2567	OP535376	OP598128	OP676087		
Cladosporium heteropogonicola		BRIP 72465a [T]	OL307932	OL332743	OL332742		
Cladosporium hillianum		CBS 125988 [T]	HM148097	HM148586	HM148341		
Cladosporium inversicolor		CBS 401.80 [T]	HM148101	HM148590	HM148345		
Cladosporium ipereniae		CBS 140483 [T]	KT600394	KT600589	KT600491		
Cladosporium iranicum		CBS 126346 [T]	HM148110	HM148599	HM148354		
Cladosporium kenpeggii		CPC 19248	KY646222	KY646225	KY646228		
Cladosporium lentulum		FMR 16288 [T]	LR813203	LR813209	LR813215		
Cladosporium licheniphilum		CBS 125990 [T]	HM148111	HM148600	HM148355		
Cladosporium longicatenatum		CBS 140485 [T]	KT600403	KT600598	KT600500		
Cladosporium lycoperdinum		CBS 126347	HM148112	HM148601	HM148356		
Cladosporium lycoperdinum		CBS 574.78C	HM148115	HM148604	HM148359		

Cladosporium macadamiae		BRIP 72269a T	MZ303810	MZ344214	MZ344195		
Cladosporium macadamiae		BRIP 72287a	MZ303811	MZ344215	MZ344196		
Cladosporium magnoliigena		MFLUCC 18-1559 T	MK347813	-	MK340864		
Cladosporium montecillanum		CPC 15605	KT600407	KT600603	KT600505		
Cladosporium montecillanum		CBS 140486 T	KT600406	KT600602	KT600504		
Cladosporium myrtacearum		CBS 126350 T	HM148117	HM148606	HM148361		
Cladosporium myrtacearum		CBS 126349	MH863925	HM148605	HM148360		
Cladosporium neapolitanum		MgPo1 T	MK387890	MK416051	MK416094		
Cladosporium needhamense		CBS 143359 T	MF473142	MF473991	MF473570		
Cladosporium neopsychrotolerans		CGMCC 3.18031 T	KX938383	KX938366	KX938400		
Cladosporium oxysporum		CBS 125991 T	NR_152267	HM148607	HM148362		
Cladosporium oxysporum		CBS 126351	MH863927	HM148608	HM148363		
Cladosporium paracladosporioides		CBS 171.54 T	HM148120	HM148609	HM148364		
Cladosporium parapenidielloides		CBS 140487 T	KT600410	KT600606	KT600508		
Cladosporium perangustum		CBS 125996 T	HM148121	HM148610	HM148365		
Cladosporium phaenocomae		CBS 128769 T	JF499837	JF499881	JF499875		

Cladosporium phyllactiniicola		CBS 126355 T	NR_111537	HM148642	HM148397		
Cladosporium phyllophilum		CBS 125992 T	HM148154	HM148643	HM148398		
Cladosporium pini-ponderosae		CBS 124456 T	FJ936160	FJ936167	FJ936164		
Cladosporium polonicum		Th/lg/2334 T	MK387894	MK416055	MK416098		
Cladosporium proteacearum		BRIP 72301a T	MZ303809	MZ344213	MZ344194		
Cladosporium pseudochalastosporoides		CBS 140490 T	NR_152296	KT600611	KT600513		
Cladosporium puris		COAD 2487 T	MK253337	MK249980	MK293777		
Cladosporium queenslandicum		BRIP 72447a T	OL307928	OL332736	OL332735		
Cladosporium rectoides		CBS 125994 T	HM148193	HM148683	HM148438		
Cladosporium rectoides		CBS 126357	MH863933	HM148684	HM148439		
Cladosporium rubrum		CMG 28	MN053018	MN066639	MN066644		
Cladosporium ruguloflflabelliforme		CBS 140494 T	KT600458	KT600655	KT600557		
Cladosporium rugulovarians		CBS 140495 T	KT600459	KT600656	KT600558		
Cladosporium scabrellum		CBS 126358 T	HM148195	HM148685	HM148440		
Cladosporium silenes		CBS 109082 T	EF679354	EF679506	EF679429		
Cladosporium sinuatum		CGMCC 3.18096 T	KX938385	KX938368	KX938402		
Cladosporium		CBS 146978 T	MZ064420	MZ078146	MZ078223		

stipagrostidicola							
Cladosporium subuliforme		CBS 126500 [T]	NR_119854	HM148686	HM148441		
Cladosporium subuliforme		CPC 15833	KT600453	KT600650	KT600552		
Cladosporium tenuissimum		CBS 125995 [T]	HM148197	HM148687	HM148442		
Cladosporium tianshanense		CGMCC 3.18033 [T]	KX938381	KX938364	KX938398		
Cladosporium uredinicola		CPC 5390	AY251071	HM148712	HM148467		
Cladosporium uwebrauniana		DTO 072-D8	MF473306	MF474156	MF473729		
Cladosporium uwebraunianum		DTO 305-H9	MF473307	MF474157	MF473730		
Cladosporium varians		CBS 126362 [T]	HM148224	HM148715	HM148470		
Cladosporium verrucocladosporioides		CBS 126363 [T]	HM148226	HM148717	HM148472		
Cladosporium vicinum		CPC 22316	MF473311	MF474161	MF473734		
Cladosporium vignae		CBS 121.25	HM148227	HM148718	HM148473		
Cladosporium welwitschiicola		CPC 18648 [T]	NR_152308	KY646226	KY646229		
Cladosporium westerdijkiae		CBS 113746 [T]	HM148061	HM148548	HM148303		
Cladosporium xanthochromaticum		CBS 140691 [T]	LN834415	LN834599	LN834511		
Cladosporium xanthochromaticum		CBS 126364	HM148122	HM148611	HM148366		
Cladosporium xylophilum		CBS 125997 [T]	NR_111541	HM148721	HM148476		
Cladosporium xylophilum		CBS 113749	HM148228	HM148719	HM148474		

Cladosporium yunnanensis		KUN HKAS 121704 ^{T}	OK338502	OL466937	OL825680		
Cladosporium longissimum		CBS 300.96 ^{T}	DQ780352	EF101385	EU570259		
Cladosporium sphaerospermum		CBS 193.54 ^{T}	DQ780343	EU570269	EU570261		
**Species**	**Previous species name**	**Culture collection number**	**ITS**	***gapdh***	***chs***	***act***	***tub***
Colletotrichum abscissum		COAD 1877 ^{T}	KP843126	KP843129	KP843132	KP843141	KP843135
Colletotrichum acerbum		CBS 128530 ^{T}	JQ948459	JQ948790	JQ949120	JQ949780	JQ950110
Colletotrichum acutatum		CBS 112996 ^{T}	JQ005776	JQ948677	JQ005797	JQ005839	JQ005860
Colletotrichum acutatum		CBS 979.69	JQ948400	JQ948731	JQ949061	JQ949721	JQ950051
Colletotrichum arboricola		CBS 144795 ^{T}	MH817944	MH817950		MH817956	MH817962
Colletotrichum australe		CBS 116478 ^{T}	JQ948455	JQ948786	JQ949116	JQ949776	JQ950106
Colletotrichum australe		CBS 131325	JQ948456	JQ948787	JQ949117	JQ949777	JQ950107
Colletotrichum brisbanense		CBS 292.67 ^{T}	JQ948291	JQ948621	JQ948952	JQ949612	JQ949942
Colletotrichum cairnsense		BRIP 63642 ^{T}	KU923672	KU923704	KU923710	KU923716	KU923688
Colletotrichum carthami		SAPA 100011 ^{T}	AB696998				AB696992
Colletotrichum chrysanthemi		IMI 364540	JQ948273	JQ948603	JQ948934	JQ949594	JQ949924
Colletotrichum cosmi		CBS 853.73 ^{T}	JQ948274	JQ948604	JQ948935	JQ949595	JQ949925
Colletotrichum costaricense		CBS 330.75 ^{T}	JQ948180	JQ948510	JQ948841	JQ949501	JQ949831
Colletotrichum coslaricense		CBS 211.78	JQ948181	JQ948511	JQ948842	JQ949502	JQ949832
Colletotrichum cuscutae		IMI 304802 ^{T}	JQ948195	JQ948525	JQ948856	JQ949516	JQ949846
Colletotrichum eriobotryae		GLMC 1935 ^{T}	MF772487	MF795423	MN191653	MN191648	MF795428

Colletotrichum fioriniae		IMI 363003	JQ948339	JQ948669	JQ949000	JQ949660	JQ949990
Colletotrichum fioriniae		CBS 128517 T	JQ948292	JQ948622	JQ948953	JQ949613	JQ949943
Colletotrichum fioriniae		CBS 129948	JQ948344	JQ948674	JQ949005	JQ949665	JQ949995
Colletotrichum fioriniae		CBS 119293	JQ948314	JQ948644	JQ948975	JQ949635	JQ949965
Colletotrichum fioriniae		**JZB330439**	**PQ570573**	**PQ573088**	**PQ573090**	**PQ573092**	**PQ573094**
Colletotrichum fioriniae		**JZB330440**	**PQ570574**	**PQ573089**	**PQ573091**	**PQ573093**	**PQ573095**
Colletotrichum godetiae		CBS 133.44 T	JQ948402	JQ948733	JQ949063	JQ949723	JQ950053
Colletotrichum godetiae		CBS 126522	JQ948411	JQ948742	JQ949072	JQ949732	JQ950062
Colletotrichum guajavae		IMI 350839 T	JQ948270	JQ948600	JQ948931	JQ949591	JQ949921
Colletotrichum indonesiense		CBS 127551 T	JQ948288	JQ948618	JQ948949	JQ949609	JQ949939
Colletotrichum jamnense		CBS 144963 T	MH846576	MH846572	MH846573	MH846575	MH846574
Colletotrichum johnstonii		CBS 128532 T	JQ948444	JQ948774	JQ949105	JQ949765	JQ950095
Colletotrichum johnstonii		IMI 357027	JQ948443	JQ948773	JQ949104	JQ949764	JQ950094
Colletotrichum kinghornii		CBS 198.35 T	JQ948454	JQ948784	JQ949115	JQ949775	JQ950105
Colletotrichum kniphofiae		CBS 143496 T	MH107884	MH107998	MH107990	MH107975	MH108037
Colletotrichum laticiphilum		CBS 112989 T	JQ948289	JQ948619	JQ948950	JQ949610	JQ949940
Colletotrichum laticiphilum		CBS 129827	JQ948290	JQ948620	JQ948951	JQ949611	JQ949941
Colletotrichum lauri		MFLUCC 17-0205 T	KY514347	KY514344	KY514341	KY514338	KY514350
Colletotrichum limetticola		CBS 114.14 T	JQ948193	JQ948523	JQ948854	JQ949514	JQ949844
Colletotrichum lupini		CBS 109225 T	JQ948155	JQ948485	JQ948816	JQ949476	JQ949806
Colletotrichum lupini		CBS 466.76	JQ948160	JQ948490	JQ948821	JQ949481	JQ949811

Colletotrichum melonis		CBS 159.84 T	JQ948194	JQ948524	JQ948855	JQ949515	JQ949845
Colletotrichum miaoliense		NTUCC 20-001-1 T	MK908419	MK908470	MK908522	MK908573	MK908624
Colletotrichum nymphaeae		ZJUC42	KC293582	KC293742	KY856139	KY855974	KC293662
Colletotrichum nymphaeae		CBS 515.78 T	JQ948197	JQ948527	JQ948858	JQ949518	JQ949848
Colletotrichum paranaense		CBS 134729 T	KC204992	KC205026	KC205043	KC205077	KC205060
Colletotrichum paxtonii		IMI 165753 T	JQ948285	JQ948615	JQ948946	JQ949606	JQ949936
Colletotrichum paxtonii		CBS 502.97	JQ948286	JQ948616	JQ948947	JQ949607	JQ949937
Colletotrichum phormii		CBS 118194 T	JQ948446	JQ948777	JQ949107	JQ949767	JQ950097
Colletotrichum phormii		CBS 199.35	JQ948447	JQ948778	JQ949108	JQ949768	JQ950098
Colletotrichum pyricola		CBS 128531 T	JQ948445	JQ948776	JQ949106	JQ949766	JQ950096
Colletotrichum rhombiforme		CBS 129953 T	JQ948457	JQ948788	JQ949118	JQ949778	JQ950108
Colletotrichum rhombiforme		CBS 131322	JQ948458	JQ948789	JQ949119	JQ949779	JQ950109
Colletotrichum roseum		CBS 145754 T	MK903611	MK903603		MK903604	MK903607
Colletotrichum salicis		CBS 607.94 T	JQ948460	JQ948791	JQ949121	JQ949781	JQ950111
Colletotrichum salicis		CBS 191 .56	JQ948461	JQ948792	JQ949122	JQ949782	JQ950112
Colletotrichum schimae		LC13880 T	MZ595885	MZ664105	MZ799347	MZ664183	MZ674003
Colletotrichum schimae		LC13881	MZ595887	MZ664106	MZ799348	MZ664185	MZ674005
Colletotrichum scovillei		CBS 126529 T	JQ948267	JQ948597	JQ948928	JQ949588	JQ949918
Colletotrichum scovillei		CBS 126530	JQ948268	JQ948598	JQ948929	JQ949589	JQ949919
Colletotrichum scovillei		CBS 120708	JQ948269	JQ948599	JQ948930	JQ949590	JQ949920
Colletotrichum simmondsii		CBS 122122 T	JQ948276	JQ948606	JQ948937	JQ949597	JQ949927
Colletotrichum simmondsii		CBS 295.67	JQ948278	JQ948608	JQ948939	JQ949599	JQ949929

Species	Previous species name	Culture collection number	ITS	*tef*	*tub2*	*cal*	*his*
Colletotrichum sloanei		IMI 364297 [T]	JQ948287	JQ948617	JQ948948	JQ949608	JQ949938
Colletotrichum subsalicis		LC13863 [T]	MZ852849		MZ799346	MZ664128	MZ673953
Colletotrichum tamarilloi		CBS 129814 [T]	JQ948184	JQ948514	JQ948845	JQ949505	JQ949835
Colletotrichum tamarilloi		CBS 129811	JQ948185	JQ948515	JQ948846	JQ949506	JQ949836
Colletotrichum walleri		CBS 125472 [T]	JQ948275	JQ948605	JQ948936	JQ949596	JQ949926
Colletotrichum wanningense		CGMCC 3.18936 [T]	MG830462	MG830318	MG830302	MG830270	MG830286
Colletotrichum orchidophilum		CBS 632.80 [T]	JQ948151	JQ948481	JQ948812	JQ949472	JQ949802
**Species**	**Previous species name**	**Culture collection number**	**ITS**	***tef***	***tub2***	***cal***	***his***
Diaporthe acutispora		CGMCC 3.18285 [T]	KX986764	KX999155	KX999195	KX999274	N/A
Diaporthe anacardii		CBS 720.97 [T]	KC343024	KC343750	KC343992	KC343266	KC343508
Diaporthe anacardii	*Diaporthe nebulae*	PMM1681	KY511337	MH708552	KY511369	N/A	N/A
Diaporthe anacardii	*Diaporthe nebulae*	Phom240	KY511315	MH708543	KY511346	N/A	N/A
Diaporthe anacardii	*Diaporthe phillipsii*	CAA817	MK792305	MK828076	MN000351	MK883831	MK871445
Diaporthe anacardii	*Diaporthe phillipsii*	CAA818	MK792307	MK828078	MN000352	MK883833	MK871447
Diaporthe anacardii	*Diaporthe portugallica*	CPC 34247	MH063905	MH063911	MH063917	MH063893	MH063899
Diaporthe anacardii	*Diaporthe*	CPC 34248	MH063906	MH063912	MH063918	MH063894	MH063900

	portugallica						
Diaporthe anacardii	*Diaporthe velutina*	CGMCC 3.18286	KX986790	KX999182	KX999223	N/A	KX999261
Diaporthe anacardii	*Diaporthe velutina*	LC4419	KX986789	KX999181	KX999222	KX999286	KX999260
Diaporthe anacardii		**JZB320308**	**PQ569442**	**PQ573096**	**PQ573097**		**PQ573098**
Diaporthe arecae		CBS 161.64 T	KC343032	KC343758	KC344000	KC343274	KC343516
Diaporthe arecae		CBS 535.75	KC343033	KC343759	KC344001	KC343275	KC343517
Diaporthe aseana		MFLUCC 12-0299a T	KT459414	KT459448	KT459432	KT459464	N/A
Diaporthe biconispora		CGMCC 3.17252 T	KJ490597	KJ490476	KJ490418	MT227578	KJ490539
Diaporthe biconispora		CGMCC 3.17251	KJ490596	KJ490475	KJ490417	N/A	KJ490538
Diaporthe camelliae-sinensis		SAUCC194.92 T	MT822620	MT855932	MT855817	MT855699	MT855588
Diaporthe camelliae-sinensis		SAUCC194.103	MT822631	MT855943	MT855828	MT855710	MT855599
Diaporthe canthii		CBS 132533 T	JX069864	KC843120	KC843230	KC843174	N/A
Diaporthe chamaeropis		CBS 454.81 T	KC343048	KC343774	KC344016	KC343290	KC343532
Diaporthe chamaeropis		CBS 753.70	KC343049	KC343775	KC344017	KC343291	KC343533
Diaporthe chamaeropis	*Diaporthe cytosporella*	FAU461	KC843307	KC843116	KC843221	KC843141	MF418283
Diaporthe cinerascens		CBS 719.96	KC343050	KC343776	KC344018	KC343292	KC343534
Diaporthe cissampeli		CBS 141331 T	KX228273	N/A	KX228384	N/A	KX228366
Diaporthe conorum		UAS002 T	FJ158120	N/A	N/A	N/A	N/A

Diaporthe corylicola		CFCC 53986 [T]	MW839880	MW815894	MW883977	MW836684	MW836717
Diaporthe decedens		CBS 109772	KC343059	KC343785	KC344027	KC343301	KC343543
Diaporthe decedens		CBS 114281	KC343060	KC343786	KC344028	KC343302	KC343544
Diaporthe elaeagni-glabrae		CGMCC 3.18287 [T]	KX986779	KX999171	KX999212	KX999281	KX999251
Diaporthe elaeagni-glabrae		LC4806	KX986780	KX999172	KX999213	KX999282	KX999252
Diaporthe foeniculina	*Diaporthe baccae*	CBS 136972	KJ160565	KJ160597	N/A	N/A	MF418264
Diaporthe foeniculina	*Diaporthe baccae*	CPC 20585	KJ160564	KJ160596	N/A	N/A	N/A
Diaporthe foeniculina	*Diaporthe rumicicola*	MFLUCC18-0739	MH846233	MK049554	MK049555	N/A	N/A
Diaporthe foeniculina	*Diaporthe rumicicola*	JZB320006	MK066126	MK078545	MK078546	N/A	N/A
Diaporthe foeniculina		CBS 111553 [T]	KC843295	KC843104	KC843209	KC843129	N/A
Diaporthe foeniculina		CBS 123208	KC343104	KC343830	KC344072	KC343346	KC343588
Diaporthe foeniculina		CBS 129528	JF951146	KC843100	KC843205	KC843124	N/A
Diaporthe foeniculina	*Diaporthe nigra*	JZB320170	MN653009	MN892277	MN887113	N/A	N/A
Diaporthe foeniculina	*Diaporthe ravennica*	MFLUCC 15-0479	KU900335	KX365197	KX432254	N/A	N/A
Diaporthe foeniculina	*Diaporthe ravennica*	MFLUCC 17-1029	KY964191	KY964147	KY964075	N/A	N/A
Diaporthe foeniculina	*Diaporthe zaobaisu*	CGMCC 3.19598	MK626922	MK654855	MK691245	N/A	MK726207
Diaporthe foeniculina	*Diaporthe zaobaisu*	PSCG032	MK626923	MK654856	MK691246	N/A	MK726208

Diaporthe forlicesenica		MFLUCC 17-1015 [T]	KY964215	KY964171	KY964099	N/A	N/A
Diaporthe hickoriae		CBS 145.26 [T]	KC343118	KC343844	KC344086	KC343360	KC343602
Diaporthe hongkongensis		CBS 115448 [T]	KC343119	KC343845	KC344087	KC343361	KC343603
Diaporthe hongkongensis		LC3079	KP267851	KP267925	KP293431	N/A	KP293505
Diaporthe isoberliniae		CPC 22549 [T]	KJ869133	N/A	KJ869245	N/A	N/A
Diaporthe maytenicola		CPC 21896 [T]	KF777157	N/A	KF777250	N/A	N/A
Diaporthe melastomatis		SAUCC194.55 [T]	MT822583	MT855896	MT855780	MT855664	MT855551
Diaporthe melastomatis		SAUCC194.80	MT822608	MT855920	MT855805	MT855687	MT855576
Diaporthe multiguttulata		CGMCC 3.17258 [T]	KJ490633	KJ490512	KJ490454	N/A	KJ490575
Diaporthe multiguttulata		CFCC 53099	MK573958	MK574633	MK574653	MK574593	MK574613
Diaporthe oncostoma		CBS 589.78	KC343162	KC343888	KC344130	KC343404	KC343646
Diaporthe oncostoma		CBS 100454	KC343160	KC343886	KC344128	KC343402	KC343644
Diaporthe parapterocarpi		CPC 22729 [T]	KJ869138	N/A	KJ869248	N/A	N/A
Diaporthe parva		CGMCC 3.19599 [T]	MK626919	MK654858	MK691248	N/A	MK726210
Diaporthe parva		PSCG035	MK626920	MK654859	MK691249	MK691169	MK726211
Diaporthe poincianellae		URM 7932 [T]	MH989509	MH989538	MH989537	MH989540	MH989539
Diaporthe psoraleae		CBS 136412 [T]	KF777158	KF777245	KF777251	N/A	N/A
Diaporthe pterocarpi	*Diaporthe pseudoinconspicua*	URM 7873	MH122535	MH122530	MH122521	MH122525	MH122518
Diaporthe pterocarpi	*Diaporthe pseudoinconspic*	URM 7874	MH122538	MH122533	MH122524	MH122528	MH122517

	ua						
Diaporthe pterocarpi	*Diaporthe inconspicua*	CBS 133813	KC343123	KC343849	KC344091	KC343365	KC343607
Diaporthe pterocarpi	*Diaporthe inconspicua*	URM7776	MG696772	MG710414	MG710395	MG710391	MG710410
Diaporthe pterocarpi	*Diaporthe lutescens*	SAUCC194.36	MT822564	MT855877	MT855761	MT855647	MT855533
Diaporthe pterocarpi		MFLUCC 10-0571 [T]	JQ619899	JX275416	JX275460	JX197451	N/A
Diaporthe pterocarpi		MFLUCC 10-0588	JQ619900	JX275417	JX275461	JX197452	N/A
Diaporthe pungensis		SAUCC194.112 [T]	MT822640	MT855952	MT855837	MT855719	MT855607
Diaporthe pungensis		SAUCC194.89	MT822617	MT855929	MT855814	MT855696	MT855585
Diaporthe saccarata		CBS 116311 [T]	KC343190	KC343916	KC344158	KC343432	KC343674
Diaporthe sophorae		JAC12639	MK432728	N/A	N/A	N/A	N/A
Diaporthe sticticа		CBS 370.54 [T]	KC343212	KC343938	KC344180	KC343454	KC343696
Diaporthe tanakae		MAFF 410600	AB245077	N/A	N/A	N/A	N/A
Diaporthe tanakae		MAFF 410127	AB245075	N/A	N/A	N/A	N/A
Diaporthe undulata		CGMCC 3.18293 [T]	KX986798	KX999190	KX999230	N/A	KX999269
Diaporthe undulata		LC8111	KY491546	KY491556	KY491566	N/A	N/A
Diaporthe vangueriae	*Diaporthe macintoshii*	BRIP 55064a	KJ197289	KJ197251	KJ197269	N/A	N/A
Diaporthe vangueriae		CPC 22703 [T]	KJ869137	N/A	KJ869247	N/A	N/A
Diaporthe vawdreyi		BRIP 57887a [T]	KR936126	KR936129	KR936128	N/A	N/A

Diaporthe xishuangbanica		CGMCC 3.18283 T	KX986784	KX999176	KX999217	N/A	N/A
Diaporthe xishuangbanica		CGMCC 3.18282	KX986783	KX999175	KX999216	N/A	KX999255
Diaporthe citri		CBS 135422 T	KC843311	KC843071	KC843187	KC843157	N/A
**Species**	**Previous species name**	**Culture collection number**	***tef***	***rpb2***			
Fusarium acutatum		CBS 401.97	MW402124	MW402813			
Fusarium acutatum		CBS 402.97 T	MW402125	MW402768			
Fusarium agapanthi		NRRL 54463 T	KU900630	KU900625			
Fusarium ananatum		CBS 118516 T	LT996091	LT996137			
Fusarium ananatum		CBS 118517	MN533988	MN534229			
Fusarium annulatum		CBS 258.54 T	MT010994	MT010983			
Fusarium annulatum		CBS 792.91	MW402153	MW402774			
Fusarium annulatum		CBS 127316	MW402021	MW402738			
Fusarium annulatum		CBS 135791	MW402054	MW402746			
Fusarium annulatum		CBS 140150	MW402077	MW402755			
Fusarium annulatum		CBS 140944	MW402079	MW402806			
Fusarium annulatum		**JZB3110489**	**PQ573099**	**PQ573101**			
Fusarium annulatum		**JZB3110490**	**PQ573100**	**PQ573102**			
Fusarium andiyaz		CBS 119856	MN533989	MN534286			
Fusarium andiyaz		CBS 119857 T	MN193854	LT996138			
Fusarium anthophilum		CBS 108.92	MW401965	MW402783			
Fusarium anthophilum		CBS 222.76E T	MW402114	MW402811			
Fusarium awaxy		CBS 119831	MN534056	MN534237			

Fusarium awaxy		LGMF 1930 [T]	MG839004	MK766941			
Fusarium bactridioides		CBS 100057 [T]	MN533993	MN534235			
Fusarium begoniae		CBS 403.97	MN193858	MN193886			
Fusarium begoniae		CBS 452.97 [T]	MN533994	MN534243			
Fusarium brevicatenulatum		CBS 404.97 [T]	MN533995	MN534295			
Fusarium brevicatenulatum		CBS 100196	MN193859	MN193887			
Fusarium bulbicola		CBS 220.76 [T]	KF466415	MW402767			
Fusarium chinhoyiense		NRRL 25221 [T]	MN534050	MN534262			
Fusarium circinatum		CBS 405.97 [T]	MN533997	MN534252			
Fusarium coicis		NRRL 66233 [T]	KP083251	KP083274			
Fusarium concentricum		CBS 450.97 [T]	AF160282	JF741086			
Fusarium concentricum		CBS 453.97	MN533998	MN534264			
Fusarium denticulatum		CBS 406.97	MN533999	MN534273			
Fusarium denticulatum		CBS 407.97 [T]	MN534000	MN534274			
Fusarium dlaminii		CBS 175.88	MN534002	MN534256			
Fusarium dlaminii		CBS 119860 [T]	MW401995	KU171701			
Fusarium ficicrescens		CBS 125177	MN534006	MN534281			
Fusarium ficicrescens		CBS 125178 [T]	KU604452	KT154002			
Fusarium fractiflexum		NRRL 28852 [T]	AF160288	LT575064			
Fusarium fredkrugeri		CBS 408.97	MW402126	MW402814			
Fusarium fredkrugeri		CBS 144209 [T]	LT996097	LT996147			
Fusarium fujikuroi		CBS 186.56	MW402108	MW402765			
Fusarium fujikuroi		CBS 221.76 [T]	MN534010	KU604255			

Fusarium globosum		CBS 428.97 [T]	KF466417	KF466406			
Fusarium globosum		CBS 431.97	MW402131	MW402816			
Fusarium globosum		CBS 120992	MW401998	MW402788			
Fusarium guttiforme		CBS 409.97 [T]	MT010999	MT010967			
Fusarium konzum		CBS 119849 [T]	LT996098	MW402733			
Fusarium konzum		CBS 139382	MW402071	MW402804			
Fusarium lactis		CBS 411.97 [T]	MN193862	MN534275			
Fusarium longicornicola		NRRL 52706 [T]	JF740788	JF741114			
Fusarium longicornicola		NRRL 52712	JF740794	JF741120			
Fusarium lumajangense		InaCCF872 [T]	LS479441	LS479850			
Fusarium madaense		CBS 146648	MW402095	MW402761			
Fusarium madaense		CBS 146669 [T]	MW402098	MW402764			
Fusarium mangiferae		CBS 119853	MN534016	MN534270			
Fusarium mangiferae		CBS 120994 [T]	MN534017	MN534271			
Fusarium mexicanum		NRRL 47473	GU737416	LR792615			
Fusarium mexicanum		NRRL 53147 [T]	GU737282	MN724973			
Fusarium mundagurra		RGB5717 [T]	KP083256	KP083276			
Fusarium musae		CBS 624.87 [T]	FN552086	MW402772			
Fusarium musae		NRRL 28893	FN552092	FN552114			
Fusarium napiforme		CBS 748.97 [T]	MN193863	MN534291			
Fusarium napiforme		CBS 135139	MN534019	MN534290			
Fusarium nygamai		CBS 572.94	MW402141	MW402819			
Fusarium nygamai		CBS 749.97 [T]	MW402151	EF470114			
Fusarium ophioides		CBS 118509	MN534020	MN534301			
Fusarium ophioides		CBS 118512 [T]	MN534022	MN534303			

Fusarium parvisorum		CMW 25267 [T]	KJ541060				
Fusarium phyllophilum		CBS 216.76 [T]	MN193864	KF466410			
Fusarium pilosicola		NRRL 29124 [T]	MN534055	MN534248			
Fusarium proliferatum		CBS 480.96 [T]	MN534059	MN534272			
Fusarium proliferatum		F026	MZ399213	MZ399210			
Fusarium pseudoanthophilum		CBS 414.97 [T]	MW402128				
Fusarium pseudocircinatum		CBS 449.97 [T]	AF160271	MN534277			
Fusarium pseudonygamai		CBS 417.97 [T]	AF160263	MN534285			
Fusarium ramigenum		CBS 418.97 [T]	KF466423	KF466412			
Fusarium ramigenum		CBS 526.97	MN534032	MN534292			
Fusarium sacchari		CBS 223.76 [T]	MW402115	JX171580			
Fusarium siculi		CBS 142222 [T]	LT746214	LT746327			
Fusarium sterilihyposum		NRRL 25623 [T]	MN193869	MN193897			
Fusarium subglutinans		CBS 215.76	MN534061	MN534241			
Fusarium subglutinans		CBS 747.97 [T]	MW402150	MW402773			
Fusarium succisae		CBS 219.76 [T]	AF160291	MW402766			
Fusarium sudanense		CBS 454.97 [T]	MN534037	MN534278			
Fusarium sudanense		CBS 675.94	MN534038	MN534279			
Fusarium temperatum		MUCL 52463 [T]		MW402776			
Fusarium terricola		CBS 483.94 [T]	MN534042	LT996156			
Fusarium terricola		CBS 119850	MN534041	MN534280			
Fusarium thapsinum		CBS 776.96 [T]	MN534044	MN534289			
Fusarium tjaetaba		NRRL 66243 [T]	KP083263	KP083275			

Fusarium tupiense		NRRL 53984 ^{T}	GU737404	LR792619			
Fusarium udum		NRRL 25199 ^{T}	KY498862	KY498875			
Fusarium verticillioides		CBS 125.73	MW402012	MW402791			
Fusarium verticillioides		CBS 218.76 ^{T}	MW402113				
Fusarium volatile		CBS 143874 ^{T}	LR596007	LR596006			
Fusarium werrikimbe		CBS 125535 ^{T}		MN534304			
Fusarium xylarioides		CBS 258.52 ^{T}	MN193874	HM068355			
Fusarium xylarioides		CBS 749.79	MN534049	MN534259			
Fusarium xyrophilum		NRRL 62710	MN193875	MN193903			
Fusarium xyrophilum		NRRL 62721 ^{T}	MN193877	MN193905			
Fusarium nirenbergiae		CBS 744.97	AF160312	LT575065			
**Species**	**Previous species name**	**Culture collection number**	**ITS**	***tub***	***tef***		
Neopestalotiopsis acrostichi		MFLUCC 17-1754 ^{T}	MK764272	MK764338	MK764316		
Neopestalotiopsis acrostichi		MFLUCC 17-1755	MK764273	MK764339	MK764317		
Neopestalotiopsis alpapicalis		MFLUCC 17-2544 ^{T}	MK357772	MK463545	MK463547		
Neopestalotiopsis alpapicalis		MFLUCC 17-2545	MK357773	MK463546	MK463548		
Neopestalotiopsis aotearoa		CBS 367.54 ^{T}	KM199369	KM199454	KM199526		
Neopestalotiopsis asiatica		MFLUCC 12-0286 ^{T}	JX398983	JX399018	JX399049		

Neopestalotiopsis australis		CBS 114159 T	KM199348	KM199432	KM199537		
Neopestalotiopsis brachiata		MFLUCC 17-1555 T	MK764274	MK764340	MK764318		
Neopestalotiopsis brasiliensis		COAD 2166 T	MG686469	MG692400	MG692402		
Neopestalotiopsis camelliae-oleiferae		CSUFTCC81 T	OK493585	OK562360	OK507955		
Neopestalotiopsis camelliae-oleiferae		CSUFTCC82	OK493586	OK562361	OK507956		
Neopestalotiopsis cavernicola		KUMCC 20-0269 T	MW545802	MW557596	MW550735		
Neopestalotiopsis chiangmaiensis		MFLUCC 18-0113 T		MH412725	MH388404		
Neopestalotiopsis chrysea		MFLUCC 12-0261 T	JX398985	JX399020	JX399051		
Neopestalotiopsis chrysea		MFLUCC 12-0262	JX398986	JX399021	JX399052		
Neopestalotiopsis clavispora		MFLUCC 12-0281 T	JX398979	JX399014	JX399045		
Neopestalotiopsis clavispora		MFLUCC 12-0280	JX398978	JX399013	JX399044		
Neopestalotiopsis cocoes		MFLUCC 15-0152 T	KX789687		KX789689		
Neopestalotiopsis coffeae-arabicae		HGUP4015	KF412647	KF412641	KF412644		
Neopestalotiopsis coffeae-		HGUP4019 T	KF412649	KF412643	KF412646		

arabicae							
Neopestalotiopsis cubana		CBS 600.96 T	KM199347	KM199438	KM199521		
Neopestalotiopsis cubana		CSUFTCC37	OK493583	OK562358	OK507953		
Neopestalotiopsis dendrobii		MFLUCC 14-0106 T	MK993571	MK975835	MK975829		
Neopestalotiopsis dendrobii		MFLUCC 14-0099	MK993570	MK975834	MK975828		
Neopestalotiopsis egyptiaca		CBS 140162 T	KP943747	KP943746	KP943748		
Neopestalotiopsis ellipsospora		MFLUCC 12-0283 T	JX398980	JX399016	JX399047		
Neopestalotiopsis foedans		CGMCC 3.9123 T	JX398987	JX399022	JX399053		
Neopestalotiopsis foedans		CGMCC 3.9178	JX398989	JX399024	JX399055		
Neopestalotiopsis formicidarum		CBS 362.72 T	KM199358	KM199455	KM199517		
Neopestalotiopsis formicidarum		CBS 115.83	KM199344	KM199444	KM199519		
Neopestalotiopsis hadrolaeliae		COAD2637 T	MK454709	MK465120	MK465122		
Neopestalotiopsis hispanica		CBS 147686 T	MW794107	MW802840	MW805399		
Neopestalotiopsis honoluluana		CBS 114495 T	KM199364	KM199457	KM199548		
Neopestalotiopsis honoluluana		CBS 111535	KM199363	KM199461	KM199546		
Neopestalotiopsis iberica		CSUFTCC91	OK493587	OK562362	OK507957		
Neopestalotiopsis iberica		CBS 147688 T	MW794111	MW802844	MW805402		
Neopestalotiopsis iraniensis		CBS 137768 T	OR230041	OR381098	OR380984		

Neopestalotiopsis javaensis		CBS 257.31 T	KM199357	KM199437	KM199543		
Neopestalotiopsis longiappendiculata		CBS 147690 T	MW794112	MW802845	MW805404		
Neopestalotiopsis lusitanica		CBS 147692 T	MW794110	MW802843	MW805406		
Neopestalotiopsis macadamiae		BRIP 63737c T	KX186604	KX186654	KX186627		
Neopestalotiopsis macadamiae		BRIP 63742a	KX186599	KX186657	KX186629		
Neopestalotiopsis maddoxii		BRIP 72266a T	MZ303782	MZ312675	MZ344167		
Neopestalotiopsis magna		MFLUCC 12-0652 T	KF582795	KF582793	KF582791		
Neopestalotiopsis mesopotamica		CBS 336.86 T	KM199362	KM199441	KM199555		
Neopestalotiopsis mesopotamica		CBS 299.74	KM199361	KM199435	KM199541		
Neopestalotiopsis musae		MFLUCC 15-0776T	KX789683	KX789686	KX789685		
Neopestalotiopsis natalensis		CBS 138.41 T	KM199377	KM199466	KM199552		
Neopestalotiopsis nebuloides		BRIP 66617 T	MK966338	MK977632	MK977633		
Neopestalotiopsis olumideae		BRIP 72273a T	MZ303790	MZ312683	MZ344175		
Neopestalotiopsis pandanicola		KUMCC 17-0175 T		MH412720	MH388389		
Neopestalotiopsis		URM7148-01 T	KJ792466		KU306739		

pernambucana								
Neopestalotiopsis pernambucana		URM7148-02	KJ792467		KU306740			
Neopestalotiopsis petila		MFLUCC 17-1737 T	MK764275	MK764341	MK764319			
Neopestalotiopsis petila		MFLUCC 17-1738	MK764276	MK764342	MK764320			
Neopestalotiopsis piceana		CBS 254.32	KM199372	KM199452	KM199529			
Neopestalotiopsis piceana		CBS 394.48 T	KM199368	KM199453	KM199527			
Neopestalotiopsis protearum		CBS 114178 T	JN712498	KM199463	KM199542			
Neopestalotiopsis rhapidis		GUCC 21501 T	MW931620	MW980441	MW980442			
Neopestalotiopsis rhizophorae		MFLUCC 17-1551 T	MK764277	MK764343	MK764321			
Neopestalotiopsis rhizophorae		MFLUCC 17-1550	MK764278	MK764344	MK764322			
Neopestalotiopsis rhododendri		GUCC 21504 T	MW979577	MW980443	MW980444			
Neopestalotiopsis rhododendri		GUCC 21505	MW979576	MW980445	MW980446			
Neopestalotiopsis rosae		CBS 101057 T	KM199359	KM199429	KM199523			
Neopestalotiopsis rosae		CBS 124745	KM199360	KM199430	KM199524			
Neopestalotiopsis rosicola		CFCC 51992 T	KY885239	KY885245	KY885243			
Neopestalotiopsis rosicola		CFCC 51993	KY885240	KY885246	KY885244			
Neopestalotiopsis samarangensis		CBS 115451	KM199365	KM199447	KM199556			

Neopestalotiopsis saprophytica		MFLUCC 12-0282 T	JX398982	JX399017	JX399048		
Neopestalotiopsis scalabiensis		MUM 21.34T	MW969748	MW934611	MW959100		
Neopestalotiopsis sichuanensis		CFCC 54338 T	MW166231	MW218524	MW199750		
Neopestalotiopsis sichuanensis		SM15-1C	MW166232	MW218525	MW199751		
Neopestalotiopsis sonneratiae		MFLUCC 17-1745 T	MK764279	MK764345	MK764323		
Neopestalotiopsis sonneratiae		MFLUCC 17-1744	MK764280	MK764346	MK764324		
Neopestalotiopsis steyaertii		IMI 192475 T	KF582796	KF582794	KF582792		
Neopestalotiopsis surinamensis		CBS 450.74 T	KM199351	KM199465	KM199518		
Neopestalotiopsis surinamensis		MFTU06-3	MT952581	MT957911	MT957936		
Neopestalotiopsis surinamensis		**JZB340093**	**PQ570575**	**PQ591924**	**PQ573105**		
Neopestalotiopsis surinamensis		**JZB340094**	**PQ570576**	**PQ591925**	**PQ573103**		
Neopestalotiopsis surinamensis		**JZB340095**	**PQ570577**	**PQ591926**	**PQ573104**		
Neopestalotiopsis thailandica		MFLUCC 17-1730 T	MK764281	MK764347	MK764325		
Neopestalotiopsis		MFLUCC 17-	MK764282	MK764348	MK764326		

thailandica		1731					
Neopestalotiopsis umbrinospora		MFLUCC 12-0285 T	JX398984	JX399019	JX399050		
Neopestalotiopsis vaccinii		MUM 21.36 T	MW969747	MW934610	MW959099		
Neopestalotiopsis vacciniicola		MUM 21.35 T	MW969751	MW934614	MW959103		
Neopestalotiopsis vheenae		BRIP 72293a T	MZ303792	MZ312685	MZ344177		
Neopestalotiopsis vitis		MFLUCC 15-1265 T	KU140694	KU140685	KU140676		
Neopestalotiopsis vitis		MFLUCC 15-1270	KU140699	KU140690	KU140681		
Neopestalotiopsis zakeelii		BRIP 72282a T	MZ303789	MZ312682	MZ344174		
Neopestalotiopsis zimbabwana		CBS 111495 T	JX556231	KM199456	KM199545		
Pestalotiopsis spathulata		CBS 356.86 T	KM199338	KM199423	KM199513		
Pestalotiopsis diversiseta		MFLUCC 12-0287 T	JX399009	JX399040	JX399073		

Ex-type, neo-type and epi-type cultures are marked with superscript T and sequences generated in the present study are in bold.
